# Cyclic Peptides from the Soft Coral-Derived Fungus *Aspergillus sclerotiorum* SCSIO 41031

**DOI:** 10.3390/md19120701

**Published:** 2021-12-10

**Authors:** Jieyi Long, Yaqi Chen, Weihao Chen, Junfeng Wang, Xuefeng Zhou, Bin Yang, Yonghong Liu

**Affiliations:** 1 CAS Key Laboratory of Tropical Marine Bio-Resources and Ecology, Guangdong Key Laboratory of Marine Materia Medica, South China Sea Institute of Oceanology, Chinese Academy of Sciences, Guangzhou 510301, China; longjieyi17@mails.ucas.ac.cn (J.L.); chenweihao17@mails.ucas.ac.cn (W.C.); wangjunfeng@scsio.ac.cn (J.W.); xfzhou@scsio.ac.cn (X.Z.); 2 College of Earth and Planetary Sciences, University of Chinese Academy of Sciences, Beijing 100049, China; 3 State Key Laboratory of Chemical Oncogenomics, Key Laboratory of Chemical Genomics, Peking University Shenzhen Graduate School, Shenzhen 518055, China; chenyq0316@pku.edu.cn

**Keywords:** sclerotides, lipodepsipeptide, *Aspergillus sclerotiorum*, nasopharyngeal carcinoma, AChE inhibitory

## Abstract

Three novel cyclic hexapeptides, sclerotides C–E (**1**–**3**), and a new lipodepsipeptide, scopularide I (**4**), together with a known cyclic hexapeptide sclerotide A (**5**), were isolated from fermented rice cultures of a soft coral-derived fungus: *Aspergillus sclerotiorum* SCSIO 41031. The structures of the new peptides were determined by 1D and 2D NMR spectroscopic analysis, Marfey’s method, ESIMS/MS analysis, and single crystal X-ray diffraction analysis. Scopularide I (**4**) exhibited acetylcholinesterase inhibitory activity with an IC_50_ value of 15.6 μM, and weak cytotoxicity against the human nasopharyngeal carcinoma cell line HONE-EBV with IC_50_ value of 10.1 μM.

## 1. Introduction

Marine microorganisms are generally considered to be a significant new chemical resource of secondary metabolites [1,2]. These organisms thrive in the hostile and competitive oceanic environment and produce a variety of chemically diverse and biologically active compounds [3,4,5], which have attracted great attention in biomedical research [6,7]. As a class of important metabolites from marine microorganisms, cyclic peptides generally possess a scarce molecular skeleton. Representatives include lucentamycins [8] and marthiapeptide A [9] from marine actinomycetes, sclerotiotides A-K [10], JBIR-15 [11], maribasins [12] and sclerotides A and B [13] from marine fungi. Of these representatives, sclerotides present a unique hexapeptide containing both anthranilic acid and dehydroamino acid residues, which are rarely reported in nature.

**Figure 1:**
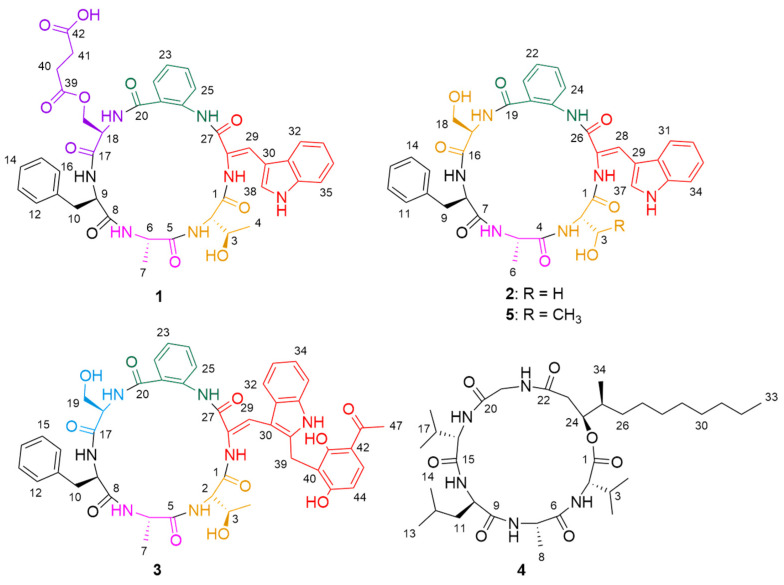
Structures of compounds **1**–**5**.

In our ongoing studies discovering structurally novel and bioactive natural hybrid peptides from soft coral-derived fungi [14], three novel cyclic hexapeptides, sclerotides C–E (**1**–**3**) and a new lipodepsipeptide scopularide I (**4**), along with a known cyclic hexapeptide, sclerotide A (**5**) [13], were obtained from *Aspergillus sclerotiorum* SCSIO 41031 (Figure 1). Herein we report the isolation, structure elucidation, and biological activities of these new cyclic peptides.

## 2. Results

### 2.1. Structure Elucidate

**Figure 2:**
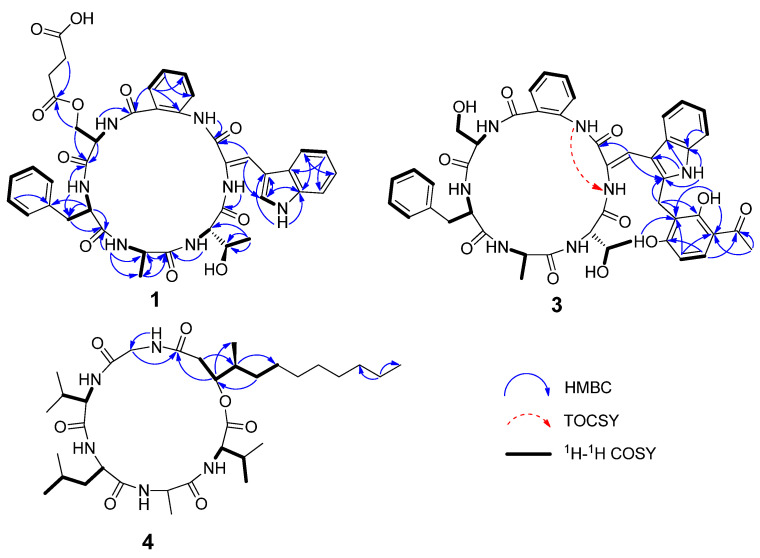
Key HMBC, ^1^H-^1^H COSY, and TOCSY correlations for **1**, **3** and **4**.

Sclerotide C (**1**) was isolated as a yellow crystal. The HRESIMS spectrum showed an ion peak at *m/z* 810.3062 [M + H]^+^, corresponding to the molecular formula C_41_H_43_N_7_O_11_, which required 24 degrees of unsaturation. Analysis of NMR spectra revealed six exchangeable amide NH protons (*δ*_H_ 7.33, 8.43, 8.74, 8.79, 8.98, 10.89) and six correlated carbonyls (*δ*_C_ 163.5, 168.8, 169.3,170.3, 171.5, 172.1, 172.6), indicating **1** was a hexapeptide. A careful analysis of the NMR data (Table 1), including HSQC, COSY, and HMBC, indicated that sclerotide C (**1**) possessed similar primary structure to sclerotide A (**5**), with the difference of four additional carbons. With respect to sclerotide A, two additional methylene groups (*δ*_H/C_ 2.45/28.6, C-40; *δ*_H/C_ 2.45/28.6, C-41) and two carbonyls (*δ*_C_ 172.1, C-39; *δ*_C_ 173.4, C-42) were easily detected from the 1D NMR and HSQC data. Moreover, the connection between C-19 and C-39 was inferred by HMBC correlections from H-19 to C-39. The downfield shifts exhibited by the Ser-NH proton (*δ*_H_ 8.98 in **1** vs. 8.62 in **5**), H-18 (*δ*_H_ 4.51 in **1** vs. 4.23 in **5**), H-19 (*δ*_H_ 4.26 and 4.31 in **1** vs. 3.70 in **5**), and C-19 (*δ*_C_ 63.0 in **1** vs. 60.9 in **5**), were all compatible with the presence of the butanedioic acid serine ester (BASE) residue in **1**. The above results revealed that compound **1** was constructed with six amino acid residues, including new esterified serine derivatives (BASE) and five known amino acids, anthranilic acid (AA), dehydrotryptophan (∆-Trp), Thr, Ala, and Phe. The connectivity between the residues of **1** was established by the key HMBC correlations (Figure 2) from NH (*δ*_H_ 8.79) of ∆-Trp to C-1 (*δ*_C_ 170.3) of Thr, NH (*δ*_H_ 7.33) of Thr to C-5(*δ*_C_ 172.6) of Ala, NH (*δ*_H_ 8.74) of Ala to C-8 (*δ*_C_ 171.5) of Phe, NH (*δ*_H_ 8.43) of Phe to C-17 (*δ*_C_ 168.8) of BASE, NH (*δ*_H_ 8.98) of BASE to C-20 (*δ*_C_ 169.3) of AA, and NH (*δ*_H_ 10.89) of AA to C-27 (*δ*_C_ 163.5) of ∆-Trp. Thus, the constitution of **1** was assigned as *cyclo* (Thr-Ala-Phe-BASE-AA-∆-Trp). The downfield chemical shift of H-29 (*δ*_H_ 7.96) implied *Z-*configuration of ∆^28,29^ in **1,** as detailed in previous research [13].

**Figure 3:**
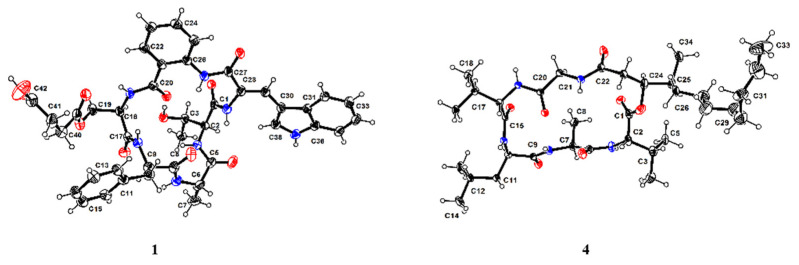
ORTEP drawing of compounds **1** and **4**.

Compound **1** was crystallized in a MeOH/H_2_O (10:1) mixture. The planar structure of **1** was further confirmed by X-ray diffraction analysis with Flack parameter 0.04(13) (Figure 3), and the unambiguous assignments of the absolute configuration as l-Thr, l-Ala, d-Phe, and d-Ser derivatives (BASE) were established. Thus, the structure of compound **1** was determined as *cyclo* (l-Thr-l-Ala-d-Phe-d-BASE-AA-*Z*-∆-Trp).

**Figure 4:**
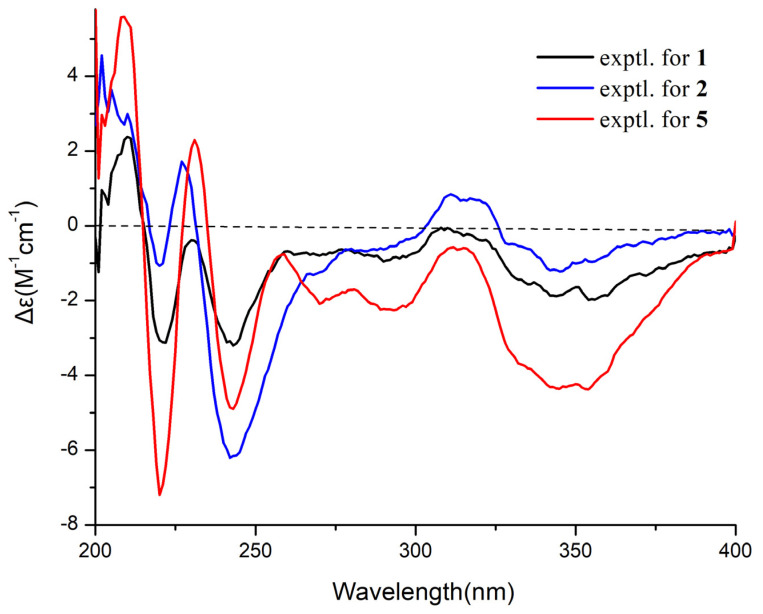
Experimental ECD spectra of compounds **1**, **2**, and **5**.

The molecular formula of sclerotide D (**2**) was determined as C_36_H_37_N_7_O_8_ based on HRESIMS data (*m*/*z* 696.2772 [M + H]^+^). Comparison of its NMR data with those of **5** indicated that **2** and **5** possessed the same carbon framework. The difference between **2** and **5** was that the threonine residue of **5** was replaced by a serine residue in the same position, which was in agreement with the 14 mass units difference. These were further confirmed by the ^1^H−^1^H COSY correlations (H-2/H-3) and the HMBC correlations (from H-3 to C-1 and C-2). Furthermore, the ECD spectra (Figure 4) of **2** were similar to those of **1** and **5**. The absolute configurations of the amino acid residues were identified as l-Ser, l-Ala, d-Phe, and d-Ser, respectively, by Marfey’s method and HPLC analysis (Supplementary Figure S44). Thus, the structure of **2** was finally established to be *cyclo* (l-Ser- l-Ala-d-Phe-d-Ser-AA-Z-∆-Trp).

**Figure 5:**
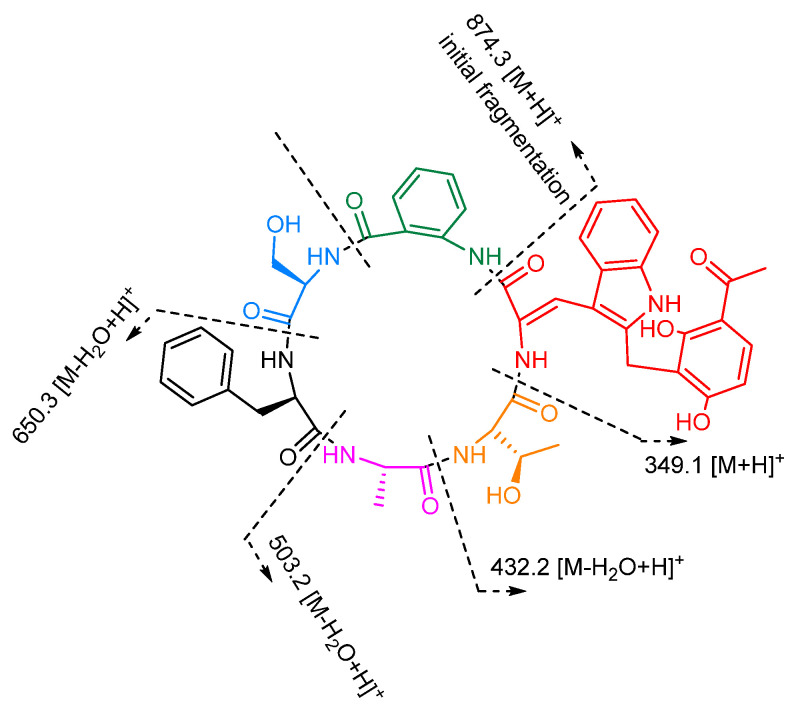
(+)-HRESIMS/MS fragments of compound **3**.

Sclerotide E (**3**) was isolated as a yellow, amorphous powder and had a molecular formula of C_46_H_47_N_7_O_11_ deduced from HRESIMS data (*m*/*z* 874.3401 [M + H]^+^), which required 27 degrees of unsaturation. The ^1^H and ^13^C NMR data of **3** (Table 2) showed similarity to those of **1**, **2**, and **5**, and the obvious differences between **3** and **5** was the additional presence of one methylene (*δ*_H/C_ 4.05/19.8, CH_2_-39), six olefins (two oxygenated) (*δ*_C_ 110.9, C-40; 162.4, C-41; 111.5, C-42; 162.8, C-45, *δ*_H/C_ 7.75/131.9, CH-43; 6.53/107.5, CH-44), and a ketone carbonyl carbon (*δ*_C_ 203.3, C-46), which suggested the presence of a tetra-substituted acetophenone group. The suggestion was proved by the ^1^H-^1^H COSY correlation between H-43 (*δ*_H_ 7.75) and H-44 (*δ*_H_ 6.53), together with the HMBC correlations (Figure 2) from H-39 to C-40, C-45, and C-41, from H-43 to C-45 and C-46, H-44 to C-40 and C-42, from CH_3_-47 to C-42 and C-46. The missing signal at *δ*_H_ 8.03 for ∆-Trp and the HMBC correlations of H-39 to C-38 revealed that the additional unit was attached to ∆-Trp at C-38. The suggested sequence was further confirmed by the ESIMS/MS spectrum of **3** (Figure 5). Consequently, the unusual amino acid residue was identified as 2-(3-acetyl-2,6-dihydroxyphenyl)-*N*-∆-acetyltryptamine (ADPAT). The downfield chemical shift of H-29 (*δ*_H_ 7.87) implied *Z*-configuration of ∆^28,29^ in **3** referred to **1**.

The connectivity between the residues of **3** was also established by the key HMBC correlations illustrated in Figure 2 and the ESIMS/MS spectrum (Figure 5). These results revealed that compound **3** consisted of *cyclo* (Thr-Ala-Phe-Ser-AA-ADPAT). Marfey’s method was employed to determine the absolute configurations of **3,** which were assigned as l-Thr, l-Ala, d-Phe, and d-Ser. Thus, the structure of **3** was established as *cyclo* [l-Thr-l-Ala-d-Phe-d-Ser-AA-Z-ADPAT].

Compound **4** was isolated as a colorless crystal, and HRESIMS data (*m/z* 652.4669 [M + H]^+^) supported a molecular formula of C_34_H_61_N_5_O_7_, accounting for 7 degrees of unsaturation. The ^1^H and ^13^C NMR data (Table 2) showed typical peptide characteristic signals: five amide NH protons (*δ*_H_ 8.65, 8.07, 8.00, 7.90, 7.42) and six ester/amide-type carbonyls (*δ*_C_ 169.1-171.9), revealing that the structure of **4** was similar to that of scopularide D [15]. Careful comparisons of the 1D and 2D NMR data between **4** and scopularide D showed that overall they were similar, but there was a slight difference which was the additional presence of one high-field methylene and the absence of a methyl attached at the long-chain fatty acid, which was in agreement with the 14 mass units difference. These were further confirmed by the ^1^H−^1^H COSY correlations (H-23/H-24/H-25/H-26/H-27) and the HMBC correlation (from H-25 to C-27). The complete structure for **4**, including absolute configurations, was confirmed by single crystal X-ray analysis (CCDC 1816072) using Cu K*α* radiation with Flack parameter of 0.05(12), which allowed the assignments of amino acid residues as l-Val, l-Ala, d-Leu, and l-Val, and gave the configurations for (24*S*,25*S*)-24-hydroxyl-25-methyllauric acid (HMLA) lipid residue (Figure 5).

### 2.2. Bioassays

The isolated compounds (**1**–**5**) were evaluated for the antimicrobial, anti-inflammatory, cytotoxic, and enzyme inhibitory activities in vitro. Compounds **1–5** did not exhibit any growth inhibition when tested against methicillin-resistant bacteria *Staphylococcus aureus*, *Staphylococcus aureus*, *Escherichia coli*, *Enterococcus faecalis*, *Pseudomonas aeruginosa*, and *Vibrio alginolyticus* or fungus *Curvularia australiensis*, *Colletotrichum acutatum*, *Fusarium oxysporum*, *Colletotrichum asianum*, and *Pyricularia oryza* in microbroth dilution assays. Likewise, **1–5** showed no inhibitory activities against lipopolysaccharide-inducted nitric oxide (NO) in RAW 264.7 cells at the concentration of 10.0 µM. In addition, the cytotoxicity against nine cancer cell lines (HL-60, K562, MOLT-4, ACHN, 786-O, OS-RC-2, THP-1, HONE1, and HONE1-EBV) were also tested. Only **4** exhibited cytotoxic toward human nasopharyngeal carcinoma (NPC) cell lines (HONE1 and HONE1-EBV) with IC_50_ values of 13.0 and 10.1 μΜ, respectively. Moreover, **4** showed moderate inhibition against acetylcholinesterase, with an IC_50_ value of 15.6 μM.

### 2.3. Molecular Docking

**Figure 6:**
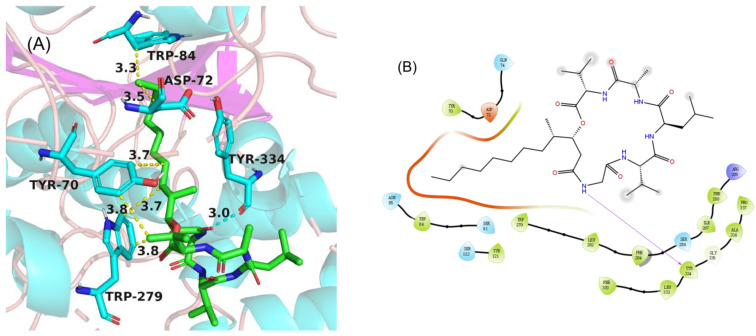
Molecular docking of **4** with AChE (PDB code: 2CMF). The binding sites of the molecule **4** (**A**) with the AChE protein. The 2D interaction details of the predicted binding mode of **4** (**B**) with the AChE.

In order to gain an insight into the molecular interactions between compounds **1**–**5** and AChE, the crystal structure of the torpedo californica acetylcholinesterase enzyme (PDB ID: 2CMF) [16] was used as the receptor, and it was subjected to an in silico molecular docking analysis with **1**–**5**, using the induced-fit module in the Schrödinger software suite. As a result, compound **4** fit comfortably into the binding pocket for alkylene-linked tacrine dimers with similar binding positions. In the 2D binding model (Figure 6B), the alkyl chain of **4** formed hydrophobic interaction with the active-site residues TRP84, ASP72, TRY70, and TRP279, and the NH of Gly formed a hydrogen bond with the active site residue TYR334. Compounds **1**–**3** and **5** were not beneficial for binding to AChE.

## 3. Materials and Methods General Experimental Procedures

### 3.1. General Experimental Procedures

Optical rotations were measured with an MCP 500 automatic polarimeter (Anton, Graz, Austria) with MeOH as a solvent. UVECD spectra were measured with a Chirascan circular dichroism spectrometer (Applied Photophysics Ltd., Surrey, UK). ^1^H, ^13^C NMR, DEPT, and 2D-NMR spectra were recorded on the Avance-700 spectrometer (Bruker, Billerica, MA, USA). HRESIMS and ESIMS spectra data were recorded on a MaXis quadrupole-time-of-flight mass spectrometer and an amaZon SL ion trap mass spectrometer (Bruker), respectively. X-ray diffraction intensity data were collected on a CrysAlis PRO CCD area detector diffractometer with graphite-monochromated Cu K*α* radiation (λ = 1.541 78 Å). Thin layer chromatography (TLC) and column chromatography (CC) were performed on plates precoated with silica gel GF 254 (10–40 µm) and over silica gel (200–300 mesh) (Qingdao Marine Chemical Factory, Qingdao, China). All solvents used were of analytical grade (Tianjin Fuyu Chemical and Industry Factory, Tianjin, China). HPLC was carried out on a Hitachi Primaide with a YMC ODS Series column (YMC-Pack ODS-A, YMC Co. Ltd., 250 × 10 mm i.d., S-5 μm, 12.0 nm, 2.0 mL/min, Kyoto, Japan) or an Angilent 1260 S3 HPLC apparatus using an ODS column (YMC-pack ODS-A, 250 × 4.6 mm, S-5 µm, 12 nm, 1.0 mL/min).

### 3.2. Fungal Material

The fungal strain SCSIO 41031 was isolated from a soft coral, which was collected in Beihai, Guangxi Province, China. The isolate was stored on Müller Hinton broth (MB) agar (malt extract 15.0 g, artificial sea salt 10.0 g, and agar 15.0 g) slants at 4.0 °C, and a voucher specimen was deposited in the CAS Key Laboratory of Tropical Marine Bio-Resources and Ecology, South China Sea Institute of Oceanology, Chinese Academy of Sciences, Guangzhou, China. Based on sequencing of the ITS region, the fungal strain SCSIO 41031 was identified as *Aspergillus sclerotiorum* with 100% similarity (GenBank no. KC478520.1).

### 3.3. Fermentation and Extraction

The strain *Aspergillus sclerotiorum* SCSIO 41031 was cultured on MB-agar plates at 25.0 °C for 7 days. The seed medium (malt extract 15.0 g and artificial sea salt 10.0 g in 1.0 L tap H_2_O, pH 7.4–7.8) was inoculated with strain SCSIO 41031 and incubated at 25.0 °C for 3 days on a rotating shaker (180 rpm). Then, a large-scale fermentation of fungal SCSIO 41031 was incubated for 30 days at room temperature in 1 L × 30 conical flasks with solid rice medium (each flask contained 200.0 g rice, 2.5 g artificial sea salt, and 250 mL H_2_O). The whole fermented cultures were overlaid and extracted with EtOAc three times to afford a brown extract (109 g).

### 3.4. Isolation and Purification

The EtOAc extract was subjected to vacuum liquid chromatography (VLC) on a silica gel column eluting with a CH_2_Cl_2_ and MeOH mixed solvent system in a step gradient (100:0−0:1, V/V) to gain nine fractions according to TLC profiles. Fr.7 (7.3 g) was divided into four parts (Frs.7-1–7-4) by ODS silica gel chromatography eluting with MeOH/H_2_O (10–100%). Fr.7-3 (1.3 g) was further purified by HPLC (42% CH_3_CN/H_2_O, 2 mL/min) to yield **5** (320.3 mg, *t*_R_ 18.5 min) and a subfraction (Fr.7-3-1, 12.2 mg, *t*_R_ 23.1 min). Moreover, Fr.7-3-1 was further purified by semipreparative HPLC (59% MeOH/H_2_O, 2 mL/min) to offer **1** (3.6 mg, *t*_R_ 26.2min) and **3** (2.1 mg, *t*_R_ 28.4 min), respectively. Fr.8 was separated by semipreparative HPLC (90% MeOH/H_2_O, 2 mL/min) to offer **4** (200.1 mg, *t*_R_ 13.5 min). Additionally, Fr.9 was divided into three subfractions (Frs. 9-1–9-3) by ODS silica gel eluting with MeOH/H_2_O (10–100%). Fr.9-3 was separated by semipreparative HPLC (40% CH_3_CN/H_2_O, 2 mL/min) to offer **2** (7.4 mg, t*_R_* 19.2 min).

Sclerotide C (**1**): yellow crystal; ${\left[a\right]}_{D}^{25}$−88 (*c* 0.06, MeOH); UV (MeOH) λ_max_ (log *ε*) 200 (4.71), 220 (4.45), 275 (3.99), 358 (4.18) nm; IR (film) ν_max_ 3325, 2926, 2358, 2331, 1683, 1541, 1456, 1207, 1141, 802, 725, 669 cm ^−1^; ^1^H and ^13^C NMR data, Table 1; HRESIMS *m/z* 810.3062 [M + H]^+^ (calculated for C_41_H_44_N_7_O_11_, 810.3093), 832.2875 [M + Na]^+^ (calculated for C_41_H_43_N_7_NaO_11_, 832.2913).

Sclerotide D (**2**): yellow solid; ${\left[a\right]}_{D}^{25}$−13 (*c* 0.09, MeOH); UV (MeOH) λ_max_ (log *ε*) 200 (4.68), 220 (4.50), 275 (3.97), 358 (4.20) nm; IR (film) ν_max_ 3298, 2929, 2854, 2362, 2330, 1681, 1521, 1456, 1236, 1203, 1139, 1049, 748, 669 cm ^−1^; ^1^H and ^13^C NMR data, Table 1; HRESIMS *m/z* 696.2772 [M + H]^+^ (calculated for C_3__6_H_38_N_7_O_8_, 696.2776), 718.2591 [M + Na]^+^ (calculated for C_36_H_37_N_7_NaO_8_, 718.2596).

Sclerotide E (**3**): yellow solid; ${\left[a\right]}_{D}^{25}$−15 (*c* 0.08, MeOH); UV (MeOH) λ_max_ (log *ε*) 200 (4.85), 280 (4.21), 352 (4.02) nm; IR (film) ν_max_ 3313, 2924, 2852, 2362, 2330, 1670, 1541,1436, 1280, 1203, 1138, 842, 802, 723 cm^−1^; ^1^H and ^13^C NMR data, Table 1; HRESIMS *m/z* 874.3401 [M + H]^+^ (calculated for C_46_H_48_N_7_O_11_, 874.3406), 896.3210 [M + Na]^+^ (calculated for C_46_H_47_N_7_NaO_11_, 896.3226).

Scopularide I (**4**): colorless needle crystal; ${\left[a\right]}_{D}^{25}$−8 (*c* 0.13, MeOH); UV (MeOH) λ_max_ (log *ε*) 200 (4.22) nm; IR (film) ν_max_ 3311, 2956, 2924, 2358, 2324, 1635, 1521, 1192, 1026 cm ^−1^; ^1^H and ^13^C NMR data, Table 1; HRESIMS *m/z* 652.4669 [M + H]^+^ (calculated for C_34_H_62_N_5_O_7_, 652.4644), 674.4478 [M + Na]^+^ (calculated for C_34_H_61_N_5_NaO_7_, 674.4463).

### 3.5. Marfey’s Analysis of ***2*** and ***3***

Compounds **2** (1.0 mg) and **3** (1.0 mg) were hydrolyzed in 1.0 mL of 6 N HCl and heated at 110 °C for 18 h. The solution was evaporated to dryness after cooled, and the hydrolysate was added: 100.0 μL of H_2_O, 100.0 μL of 1% FDAA (Marfey’s reagent, 1-fluoro-2,4-dinitrophenyl-5-L-alanine amide) in acetone, and 20.0 μL of 1 M NaHCO_3_. The mixture was kept at 40 °C for 1 h, and the reaction was quenched by adding 20.0 μL of 1 M HCl [14]. The dried mixture was dissolved in MeOH and HPLC analyses performed on an Agilent Technologies 1200 Infinity system (column: YMC-Pack ODS-A column, 250 × 4.6 mm l.D., S-5 μm, 12 nm; mobile phase: CH_3_CN/H_2_O (0.03% TFA in H_2_O), linear gradients started with 15% CH_3_CN and finished with 60% CH_3_CN in 40 min; 100% CH_3_CN from 40 min to 50 min, flow rate was 1 mL/min, with UV detection at an absorbance of 340 nm). The standard amino acids were derived with FDAA in the same process. The retention times of these standard amino acids were as follows: FDAA, t*_R_* 22.8 min, d/l-Phe, t*_R_* 33.5/30.8 min; d/l-Ala, t*_R_* 22.8/20.9 min; d/l-Thr, t*_R_* 20.1/17.1 min; d/l-Ser, t*_R_* 16.4/16.1 min. The retention times of amino acids in hydrolysate of **2** were 16.1, 16.4, 20.9, and 33.5 min, respectively. Comparison of these retention times confirmed that the amino acids in **2** were l-Ser, d-Ser, l-Ala, d-Phe (Supplementary Figure S44). The retention times of amino acids in hydrolysate of **3** were 16.4, 17.1, 20.9, and 33.5 min, respectively, which were confirmed as _d_-Ser, _l_-Thr, _l_-Ala, and _d_-Phe (Supplementary Figure S44).

### 3.6. X-ray Crystallographic Analysis

Sclerotide C (**1**) was crystallized from the mixture of methanol and H_2_O (10:1) to give yellow crystals. The crystal data was as follows: monoclinic, space group P2_1_2_1_2_1_ with *a* = 10.5314(2)Å, *b* = 12.7668(3)Å, *c* = 30.3494(8)Å, *V* = 4080.55(16)Å^3^, Z = 4, *D*_calc_ = 1.318g/cm^3^, *R* = 0.0706, *w*R_2_ = 0.1661. The absolute configuration was determined on the basis of a Flack parameter of 0.04(13). Crystallographic data (excluding structure factors) for structure **1** in this paper were deposited with the Cambridge Crystallographic Data Centre as supplementary publication number CCDC 2026267. Copies of the data can be obtained, free of charge, on application to CCDC, 12 Union Road, Cambridge CB21EZ, UK (fax: +44-(0)-1223-336033 or e-mail: deposit@ccdc.cam.ac.uk).

Scopularide I (**4**) was crystallized from the mixture of methanol and H_2_O (10:1) to give colorless crystals. The crystal data was as follows: monoclinic, space group C2 with *a* = 25.7630(3)Å, *b* = 9.31313(9)Å, *c* = 17.4956(2)Å, *V* = 3998.24(8)Å^3^, Z = 4, D_calc_ = 1.143g/cm^3^, R = 0.0920, *w*R_2_ = 0.2540. The absolute configuration was determined on the basis of a Flack parameter of -0.03(12). Crystallographic data (excluding structure factors) for structure **4** in this paper were deposited with the Cambridge Crystallographic Data Centre as supplementary publication number CCDC 2026265. Copies of the data can be obtained, free of charge, on application to CCDC, 12 Union Road, Cambridge CB21EZ, UK (fax: +44-(0)-1223-336033 or e-mail: deposit@ccdc.cam.ac.uk).

### 3.7. Bioactivity Assay

The AChE inhibition activity was measured based on the modified Ellman’s method [17]. Tacrine was used as positive drug. The inhibition rates of AChE were calculated using Origin 8.0 software.

Antibacterial activities against methicillin-resistant *Staphylococcus aureus*, *Staphylococcus aureus*, *Escherichia coli*, *Enterococcus faecalis*, *Pseudomonas aeruginosa,* and *vibrio alginolyticus* and antifungal activities against *Curvularia australiensis*, *Colletotrichum acutatum*, *Fusarium oxysporum*, *Colletotrichum asianum,* and *Pyricularia oryza* were tested using a modification of the broth microdilution method [18].

The obtained compounds (**4** and **5**) were evaluated for their cytotoxic activities against three cancer cell lines, THP-1, HONE1, and HONE1-EBV. The THP-1, HONE1, and HONE1-EBV cell lines were obtained from Sun Yat-sen University Cancer Center. The cytotoxic activity was determined by the CCK-8 (Dojindo) method [19]. Briefly, THP-1, HONE1, and HONE1-EBV cells were cultured in DMEM media supplemented with 10% phosphate-buffered saline (FBS), respectively. The cells were seeded at a density of 400 to 800 cells/well in 384-well plates and then incubated with the compounds in a gradient concentration (50.0, 10.0, 2.0, 0.4, and 0.08 μM) or with a solvent control for 72 h, followed by the addition of CCK-8 reagent. The OD value of each well was measured at 450 nm using a SpectraMax M5 Microplate Reader (Molecular Devices). Sorafenib functioned as the positive control. Dose–response curves were plotted to determine IC_50_ based upon the average values of three parallel experiments using Prism 5.0.

### 3.8. Molecular Docking Analysis

The molecular docking analysis with the structure of AChE (PDB code: 2CMF) [16] was conducted according to the procedure described previously [20].

## 4. Conclusions

In summary, the chemical investigation of the soft coral-derived fungus *Aspergillus sclerotiorum* SCSIO 41031 has led to four new compounds—three cyclic hexapeptides (**1**–**3**), and a new lipodepsipeptide (**4**). Their structures, including their absolute configurations, were determined by comprehensive spectroscopic methods and X-ray crystallographic analysis together with Marfey’s method. The folding of the peptide backbone remains to be studied. The in vitro bioassay and in silico docking study revealed compound **4** to be a potential anti-nasopharyngeal cancer drug and a moderate AChE inhibitor.

## Figures and Tables

**Table 1:** ^1^H and ^13^C NMR data for **1** and **2** (700, 175 MHz, DMSO-*d*_6_, TMS, *δ* ppm).

Position		Sclerotide C (1)		Position		Sclerotide D (2)	
		*δ*_C_	*δ*_H_ (*J* in Hz)			*δ*_C_	*δ*_H_ (*J* in Hz)
Thr	1	170.3		Ser	1	169.7	
	2	59.0	4.32, m		2	56.8	4.55, td (7.2, 4.2)
	3	65.5	4.44, m		3	60.7	4.04, dd (11.2, 8.7)
	4	20.8	1.17, d (6.4)				3.96, dd (11.3, 4.3)
	NH		7.33, d (8.4)		NH		7.13, d (8.1)
Ala	5	172.6		Ala	4	172.5	
	6	49.6	4.22, m		5	49.2	4.25, m
	7	16.7	1.28, d (7.3)		6	16.3	1.25, d (7.3)
	NH		8.74, d (6.1)		NH		8.62, d (6.5)
Phe	8	171.5		Phe	7	171.8	
	9	54.4	4.63, “q” like (7.6)		8	54.5	4.53, “q” like (7.6)
	10	35.6	2.89, dd (14.1, 7.9)		9	35.1	2.93, dd (14.0, 7.3)
			2.93, dd (14.1, 7.1)				2.88, dd (14.0, 7.9)
	11	137.6			10	137.5	
	12	129.0	7.20, d (7.4)		11	129.0	7.20, d (7.2)
	13	128.1	7.24, t (7.4)		12	128.1	7.23, m
	14	126.3	7.18, t (7.4)		13	126.2	7.17, m
	15	128.1	7.24, t (7.4)		14	128.1	7.23, m
	16	129.0	7.20, d (7.4)		15	129.0	7.20, d (7.2)
	NH		8.43, d (7.5)		NH		8.26, d (7.0)
BASE	17	168.8		Ser	16	170.8	
	18	54.4	4.51, td (7.2, 4.2)		17	59.2	4.18, q (5.8)
	19	63.0	4.26, dd, (11.3, 7.9)		18	60.8	3.70, d (5.7)
			4.31, m				
	39	172.1			NH		8.65, d (5.5)
	40	28.6	2.45, ovl ^a^				
	41	28.6	2.45, ovl ^a^				
	42	173.4					
	NH		8.98, d (6.2)				
AA	20	169.3		AA	19	169.5	
	21	122.0			20	119.6	
	22	129.2	7.79, dd (7.8, 1.1)		21	129.1	7.96, dd (8.1, 1.1)
	23	122.7	7.23, m		22	122.2	7.20, m
	24	132.2	7.59, ddd (8.2, 7.8, 1.0)		23	132.6	7.59, ddd (8.4, 6.6, 1.0)
	25	121.0	8.58, d (8.2)		24	119.6	8.79, d (8.3)
	26	138.6			25	139.7	
	NH		10.89, s		NH		11.75, s
∆-Trp	27	163.5		∆-Trp	26	163.4	
	28	122.2			27	121.8	
	29	125.9	7.96, s		28	126.3	7.97, s
	30	108.6			29	108.7	
	31	127.4			30	127.4	
	32	117.7	7.76, d (7.8)		31	117.7	7.76, d (7.7)
	33	120.4	7.16, m		32	120.4	7.16, m
	34	122.0	7.18, m		33	122.2	7.20, m
	35	112.0	7.42,d (7.9)		34	111.9	7.42, d (7.9)
	36	135.5			35	135.5	
	37-NH		11.95, d (1.9)		36-NH		11.97, d (2.0)
	38	128.7	8,00 d (2.8)		37	128.7	8.03, d (2.8)
	NH		8.79, s		NH		8.82, s

^a^ Ovl: overlapped or multiplet with other signals.

**Table 2:** ^1^H and ^13^C NMR data for **3** and **4** (700, 175 MHz, DMSO-*d*_6_, TMS, *δ* ppm).

Position		Sclerotides E (3)		Position		Scopularide I (4)	
		*δ*_C_	*δ*_H_ (*J* in Hz)			*δ*_C_	*δ*_H_ (*J* in Hz)
Thr	1	169.6		Val	1	170.8	
	2	57.5	4.35, dd (9.4, 3.2)		2	57.7	4.09, ovl ^a^
	3	66.2	4.17, td (6.5, 3.7)		3	29.6	2.07, m
	4	19.9	1.01, d (6.4)		4	18.8	0.87, ovl ^a^
	3-OH		4.80, br s		5	17.4	0.86, ovl ^a^
	NH		7.32, d (8.6)		NH		7.42, br s
Ala	5	171.9		Ala	6	171.9	
	6	48.1	4.47, m		7	48.0	4.19, m
	7	16.6	1.14, d (7.2)		8	17.5	1.22, d (7.1)
	NH		8.39, d (8.5)		NH		8.00, d (7.8)
Phe	8	170.3		Leu	9	171.4	
	9	54.4	4.55, “q” like		10	51.9	4.04, ovl ^a^
	10	35.6	2.90, dd (13.7, 7.8)		11	38.7	1.49, m
			3.02, dd (13.8, 7.5)		12	24.2	1.64, m
	11	137.6			13	23.0	0.89, ovl ^a^
	12	129.1	7.27, d (7,3)		14	21.0	0.81, ovl ^a^
	13	128.1	7.25, m		NH		8.65, d (6.6)
	14	126.3	7.2, m	Val	15	171.7	
	15	128.1	7.25, m		16	58.8	4.05, ovl ^a^
	16	129.1	7.27, d (7,3)		17	29.5	1.85, m
	NH		8.31, d (7.8)		18	19.0	0.88, ovl ^a^
Ser	17	170.8			19	19.1	0.88, ovl ^a^
	18	57.8	4.23, q (6.8)		NH		8.07, d (7.6)
	19	61.1	3.67, m	Gly	20	169.1	
	19-OH		5.15, m		21	42.0	4.08, ovl ^a^
	NH		8.31, d (7.8)				3.45, dd (17.1, 3.7)
AA	20	168.1			NH		7.90, dd (6.6, 3.9)
	21	123.1		HMLA	22	169.9	
	22	129.7	7.94, d (7.9)		23	37.5	2.53, dd (15.6, 9.6)
	23	129.2	7.27, m				2.24, m
	24	132.1	7.58, ddd (8.4, 6.6, 1.0)		24	75.5	4.91, ddd (9.4, 3.8, 2.3)
	25	122.2	8.36, m		25	36.3	1.67, m
	26	138.3			26	31.6	1.36, m
	NH		10.86, br s				1.02, m
ADPAT	27	164.2			27	26.6	1.28, ovl ^a^
	28	123.2					1.18, ovl ^a^
	29	126.2	7.87, s		28	28.7	1.23, ovl ^a^
	30	106.8			29	28.9	1.23, ovl ^a^
	31	125.3			30	29.3	1.23, ovl ^a^
	32	120.7	7.34, m		31	31.3	1.23, ovl ^a^
	33	120.9	7.02, m		32	22.1	1.27, ovl ^a^
	34	120.1	7.02, m		33	14.0	0.85, s
	35	112.6	7.34, m		34	14.8	0.83, ovl ^a^
	36	135.9					
	37-NH		10.78, s				
	38	141.6					
	NH		9.32, s				
	39	19.8	4.05, d (14.7)				
	40	110.9					
	41	162.4					
	41-OH		13.27, s				
	42	111.5					
	43	131.9	7.75, d (8.9)				
	44	107.5	6.53, d (8.8)				
	45	162.8					
	45-OH		11.01, s				
	46	203.3					
	47	26.1	2.54, s				

^a^ Ovl: overlapped or multiplet with other signals.

## Supplementary Materials

The following are available online at https://www.mdpi.com/article/10.3390/md19120701/s1, Figures S1–S43: ^1^H NMR, ^13^C NMR, DEPT, HSQC, HMBC, COSY, ROESY, TOESY, UV, HRESIMS, and CD spectra of compounds **1**−**4**. Figure S44–S45: HPLC analysis of FDAA derivatives of standard amino acids and FDAA derivatives of compound **2** and **3**. Figure S46 and Table S1: data of compound **4** against AChE. Figure S47 and Table S2: data of compound **4** against HONE1-EBV and HONE1.
