# Supplementary material for: Cyclic Peptides from the Soft Coral-Derived Fungus Aspergillus sclerotiorum SCSIO 41031
Source: Mar Drugs. 2021 Dec 10;19(12):701. doi: 10.3390/md19120701 (PMC8703611; doi:10.3390/md19120701)
Supplement: Supplementary file 1 [file marinedrugs-19-00701-s001.zip › marinedrugs-1500581-supplementary.pdf]

Sclerotides C-E and Scopularide I, New Cyclic Peptides from the Soft-Coral Derived Fungus *Aspergillus sclerotiorum*

Jieyi Long[1,2], Yaqi Chen[3], Weihao Chen[1,2], Junfeng Wang[1], Xuefeng Zhou[1], Bing Yang[1]*, and Yonghong Liu[1,2]*

[1] CAS Key Laboratory of Tropical Marine Bio-resources and Ecology/Guangdong Key Laboratory of Marine Materia Medica/Innovation Academy of South China Sea Ecology and Environmental Engineering, South China Sea Institute of Oceanology, Chinese Academy of Sciences, Guangzhou 510301, P. R. China;

[2] University of Chinese Academy of Sciences, 19 Yuquan Road, Beijing 100049, P. R. China;

[3] State Key Laboratory of Chemical Oncogenomics, Key Laboratory of Chemical Genomics, Peking University Shenzhen Graduate School, Shenzhen, 518055 China;

Contents

ITS sequence of the strain *Aspergillus sclerotiorum* SCSIO 41031 4

Figure S1. ^{1}H NMR spectrum of 1 (DMSO-d_6, 700 MHz) 5

Figure S2. ^{13}C NMR spectrum of 1 (DMSO-d_6, 175 MHz) 5

Figure S3. DEPT spectrum of 1 (DMSO-d_6) 6

Figure S4. HSQC spectrum of 1 (DMSO-d_6) 6

Figure S5. ^{1}H-^{1}H COSY spectrum of 1 (DMSO-d_6) 7

Figure S6. HMBC spectrum of 1 (DMSO-d_6) 7

Figure S7. HRESIMS spectrum of 1 8

Figure S8. IR spectrum of 1 8

Figure S9. UV spectrum of 1 9

Figure S10. CD spectrum of 1 9

Figure S11. ^{1}H NMR spectrum of 2 (DMSO-d_6, 700 MHz) 10

Figure S12. ^{13}C NMR spectrum of 2 (DMSO-d_6, 175 MHz) 10

Figure S13. DEPT spectrum of 2 (DMSO-d_6) 11

Figure S14. HSQC spectrum of 2 (DMSO-d_6) 11

Figure S15. ^{1}H-^{1}H COSY spectrum of 2 (DMSO-d_6) 12

Figure S16. HMBC spectrum of 2 (DMSO-d_6) 12

Figure S17. HRESIMS spectrum of 2 13

Figure S18. IR spectrum of 2 13

Figure S19. UV spectrum of 2 14

Figure S20. CD spectrum of 2 14

Figure S21. ^{1}H NMR spectrum of 3 (DMSO-d_6, 500 MHz) 15

Figure S22. ^{13}C NMR spectrum of 3 (DMSO-d_6, 125 MHz) 15

Figure S23. DEPT spectrum of 3 (DMSO-d_6) 16

Figure S24. HSQC spectrum of 3 (DMSO-d_6) 16

Figure S25. ^{1}H-^{1}H COSY spectrum of 3 (DMSO-d_6) 17

Figure S26. HMBC spectrum of 3 (DMSO-d_6) 17

Figure S27. TOCSY spectrum of 3 18

Figure S28. HRESIMS spectrum of 3 18

Figure S29. HRESIMS/MS fragmentation of 3 19

Figure S30. IR spectrum of 3 20

Figure S31. UV spectrum of 3 20
Figure S32. CD spectrum of 3 21
Figure S33. ^{1}H NMR spectrum of 4 (DMSO-d_6, 500 MHz) 21
Figure S34. ^{13}C NMR spectrum of 4 (DMSO-d_6, 125 MHz) 22
Figure S35. DEPT spectrum of 4 (DMSO-d_6) 22
Figure S36. HSQC spectrum of 4 (DMSO-d_6) 23
Figure S37. ^{1}H-^{1}H COSY spectrum of 4 (DMSO-d_6) 23
Figure S38. HMBC spectrum of 4 (DMSO-d_6) 24
Figure S39. NOESY spectrum of 4 (DMSO-d_6) 25
Figure S40. HRESIMS spectrum of 4 25
Figure S41. IR spectrum of 4 26
Figure S42. UV spectrum of 4 26
Figure S43. CD spectrum of 4 27
Figure S44. HPLC analysis of FDAA derivatives of standard amino acids (YMC-Pack ODS-A column, 250*4.6 mml.D., S-5 μm, 12 nm). 28
Figure S45. HPLC analysis of FDAA derivatives of compound 2 and 3 (YMC-Pack ODS-A column, 250*4.6 mml.D., S-5 μm, 12 nm) 29
Table S1.Test concentration and OD value of compound 4 against AChE 29
Figure S46. IC_{50} curve of compound 4 against AChE. 30
Table S2.Test concentration and OD value of compound 4 against HONE1-EBV and HONE1. 30
Figure S47. IC_{50} curve of compound 4 against HONE1-EBV and HONE1. 30

ITS sequence of the strain *Aspergillus sclerotiorum* SCSIO 41031

TGCGGAAGGATCATTACTGAGTGAGGGTCCCTCGGGGCCCAACCTCCCACCCGTGTAT
ACCGTACCTTGTTGCTTCGGCGGGCCCGCCGCGCAAGCGGCCGCCGGGGGGGGGCGT
CAAACCCCCCTCCCTAGGCGAGCGCCCGCCGGAGACACCAACGTGAACACTGTCTGA
AGTTTTGTTGTCTGAGTTCGATTGTATCGCAATCAGTTAAAACTTTCAACAATGGATCTC
TTGGTTCCGGCATCGATGAAGAACGCAGCGAAATGCGATAATTAATGTGAATTGCAGAA
TTCAGTGAATCATCGAGTCTTTGAACGCACATTGCACCCCCTGGTATTCCGGGGGGTAT
GCCTGTCCGAGCGTCATTGCTGCCCTCAAGCACGGCTTGTGTGTTGGGTCGTCGTCCC
CCCGGGGACGGGCCCGAAAGGCAGCGGCGGCACCGCGTCCGGTCCTCGAGCGTATGG
GGCTTTGTCACCCGCTCTTGTAGGCCCGGCCGGCGCTGGCCGACGCTGAAAAGCAACC
AACTATTTCTCCAGGTTGACCTCGGATCAGGTAGGGATACCCGCTGAACTTAAGCATAT
C

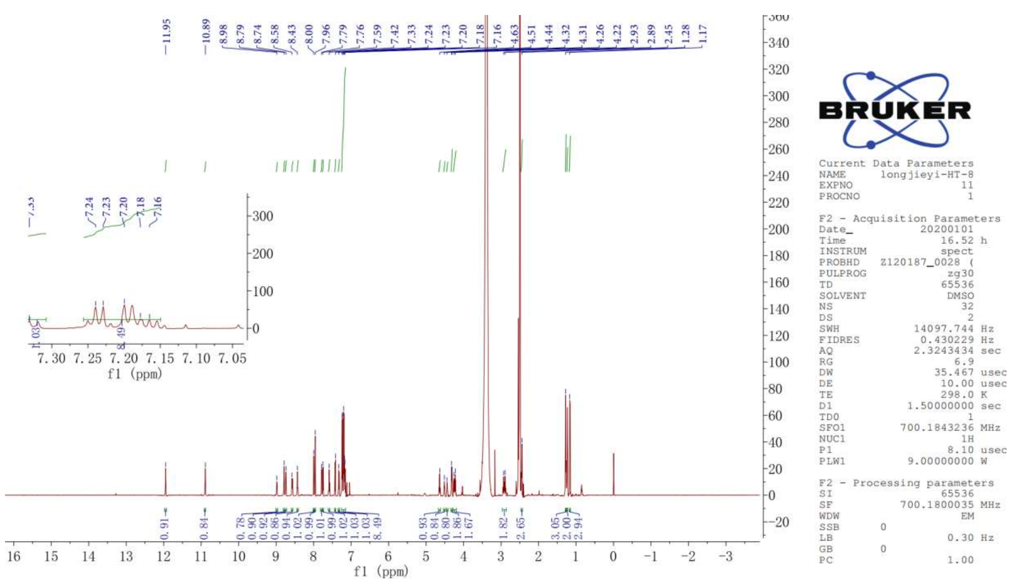

Figure S1. ^{1}H NMR spectrum of **1** (DMSO-d_6, 700 MHz)

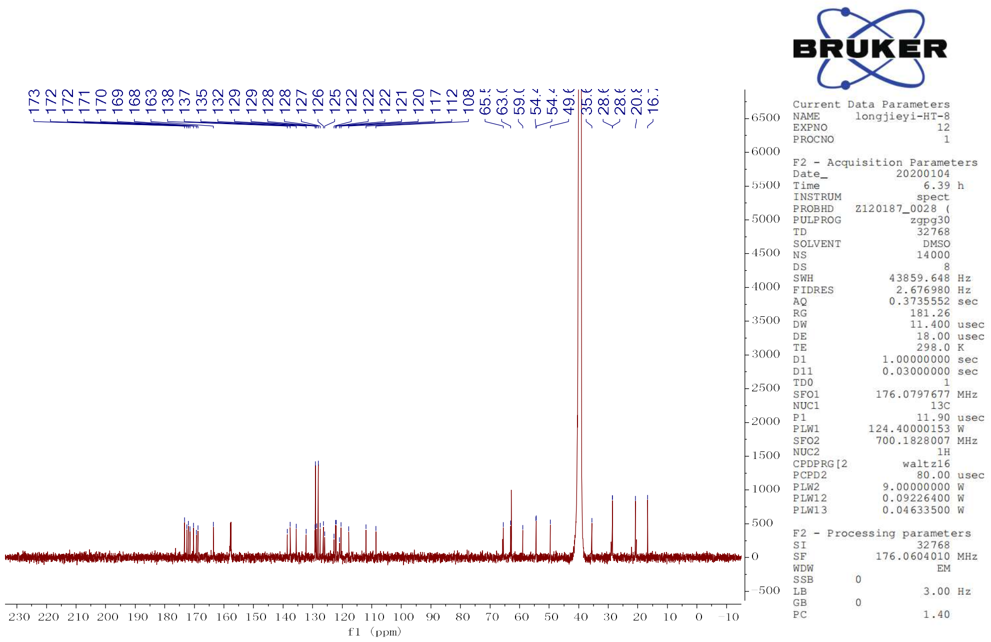

Figure S2. ^{13}C NMR spectrum of **1** (DMSO-d_6, 175 MHz)

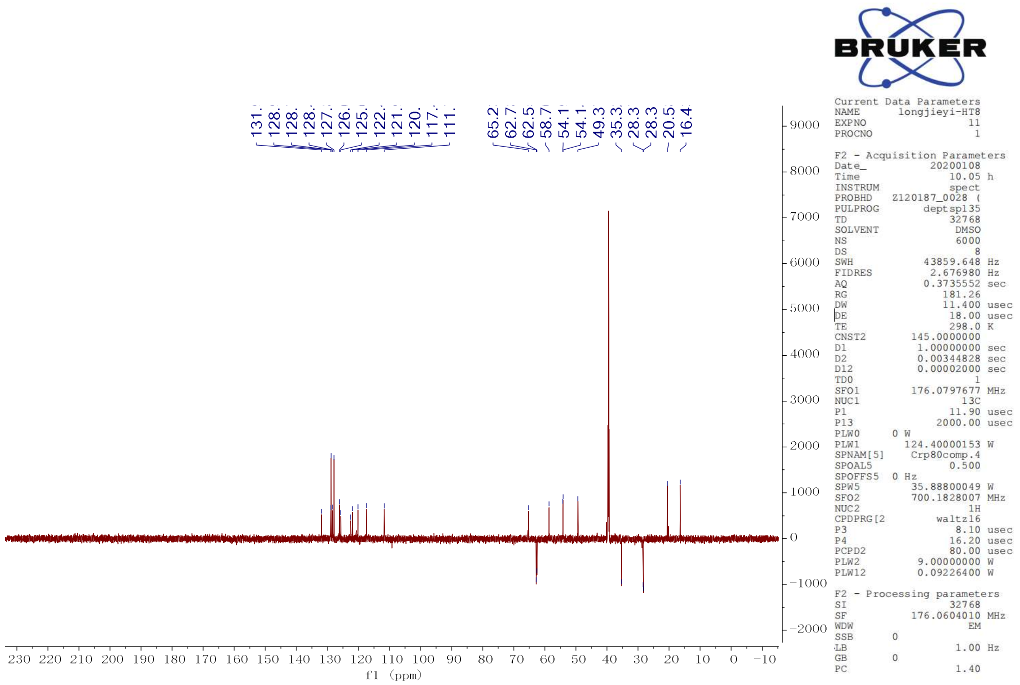

Figure S3. DEPT spectrum of **1** (DMSO-d_6)

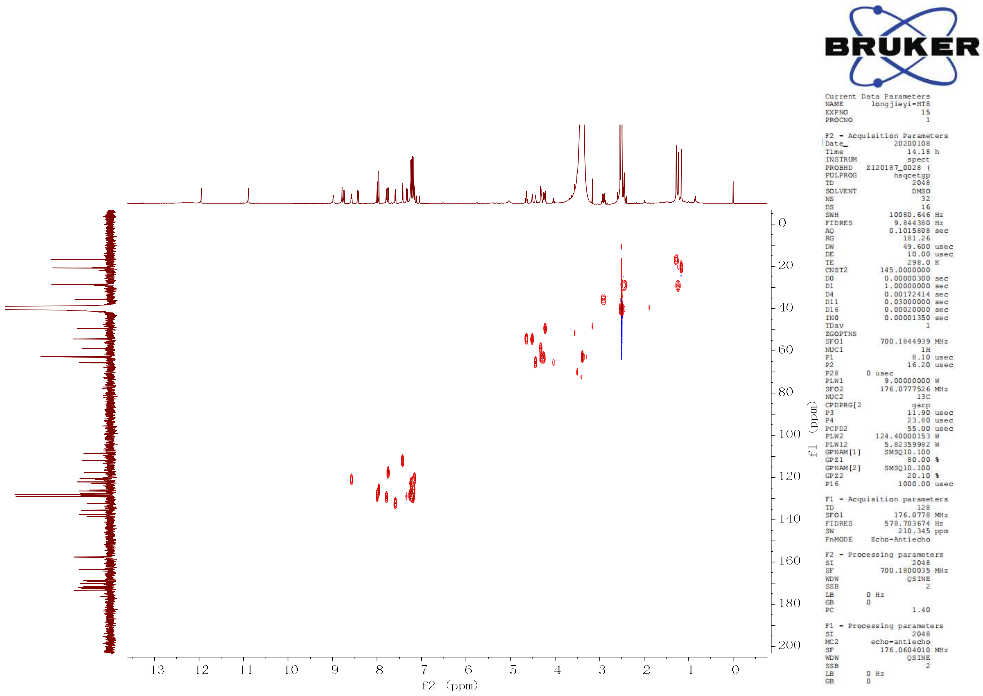

Figure S4. HSQC spectrum of **1** (DMSO-d_6)

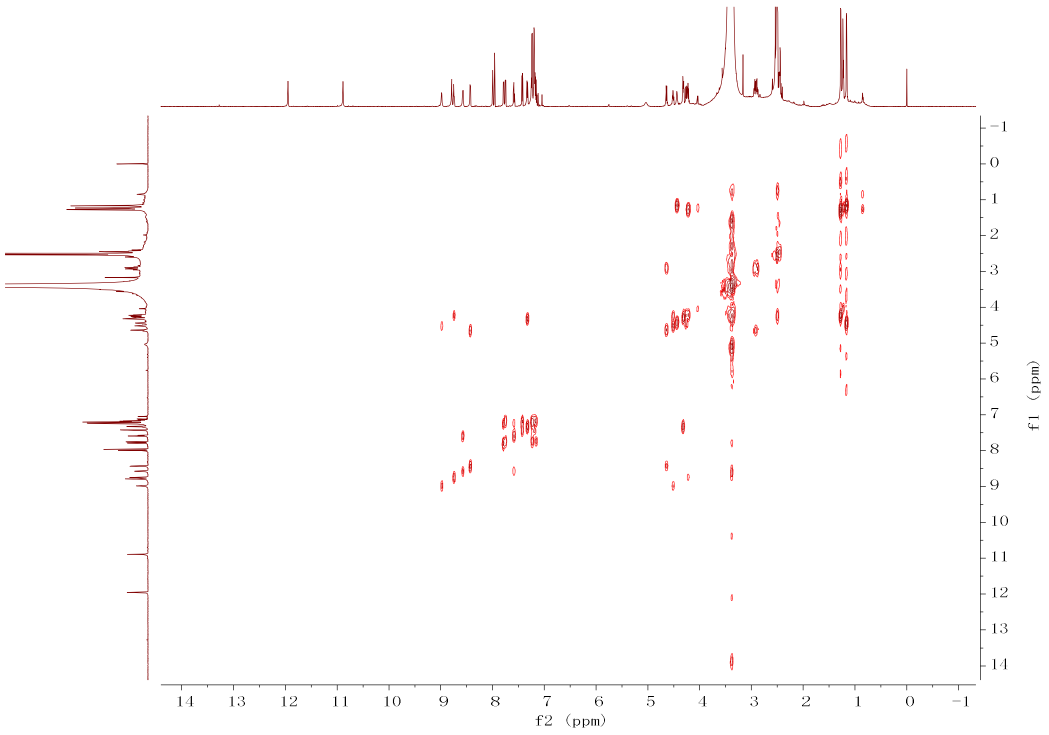

Figure S5. ^{1}H-^{1}H COSY spectrum of 1 (DMSO-d_6)

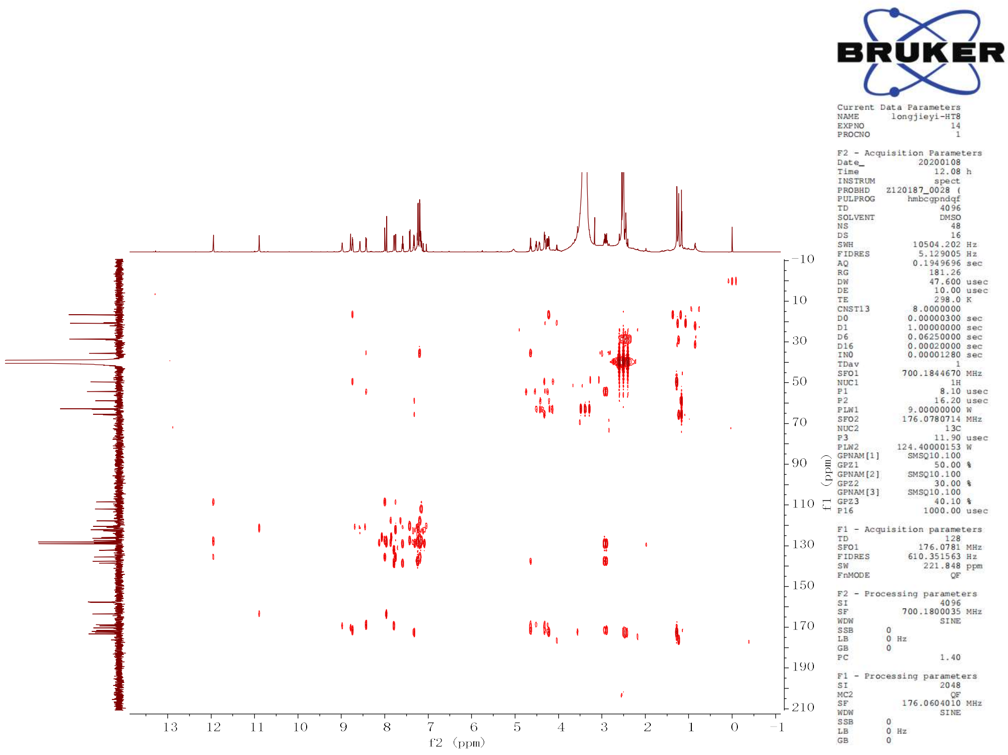

Figure S6. HMBC spectrum of **1** (DMSO-d_6)

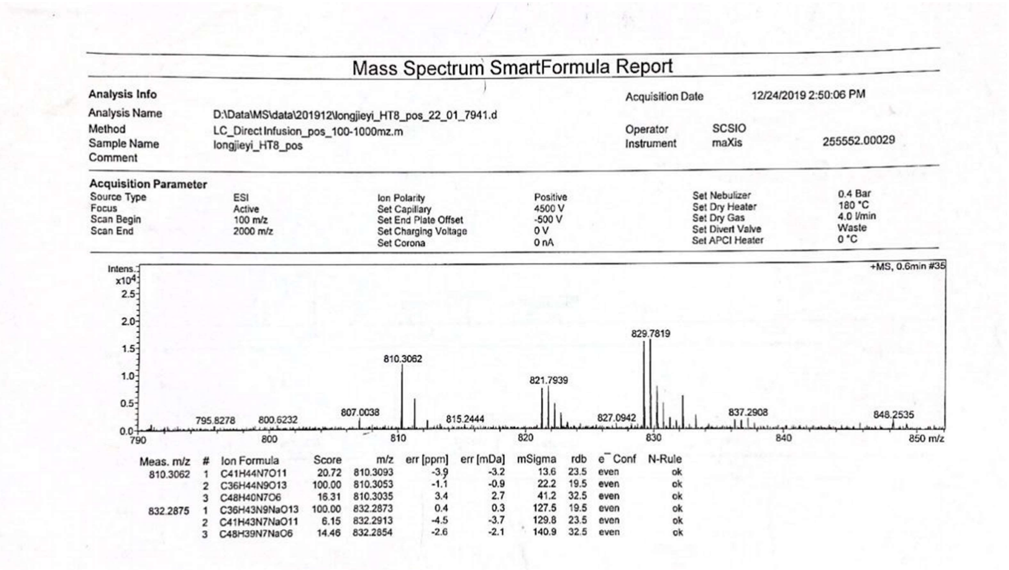

Mass Spectrum SmartFormula Report

Analysis Info

Acquisition Date 12/24/2019 2:50:06 PM

Analysis Name D:\Data\MS\data\201912\longjieyi_HT8_pos_22_01_7941.d

Method LC_Direct Infusion_pos_100-1000mz.m

Operator SCSIO

Sample Name longjieyi_HT8_pos

Instrument maXis 255552.00029

Comment

Acquisition Parameter

Source Type	ESI	Ion Polarity	Positive	Set Nebulizer	0.4 Bar
Focus	Active	Set Capillary	4500 V	Set Dry Heater	180 °C
Scan Begin	100 m/z	Set End Plate Offset	-500 V	Set Dry Gas	4.0 l/min
Scan End	2000 m/z	Set Charging Voltage	0 V	Set Divert Valve	Waste
		Set Corona	0 nA	Set APCI Heater	0 °C

Meas. m/z	#	Ion Formula	Score	m/z	err [ppm]	err [mDa]	mSigma	rdb	e⁻ Conf	N-Rule
810.3062	1	C41H44N7O11	20.72	810.3093	-3.9	-3.2	13.6	23.5	even	ok
	2	C36H44N9O13	100.00	810.3053	-1.1	-0.9	22.2	19.5	even	ok
	3	C48H40N7O6	16.31	810.3035	3.4	2.7	41.2	32.5	even	ok
832.2875	1	C36H43N9NaO13	100.00	832.2873	0.4	0.3	127.5	19.5	even	ok
	2	C41H43N7NaO11	6.15	832.2913	-4.5	-3.7	129.8	23.5	even	ok
	3	C48H39N7NaO6	14.46	832.2854	-2.6	-2.1	140.9	32.5	even	ok

Figure S7. HRESIMS spectrum of **1**

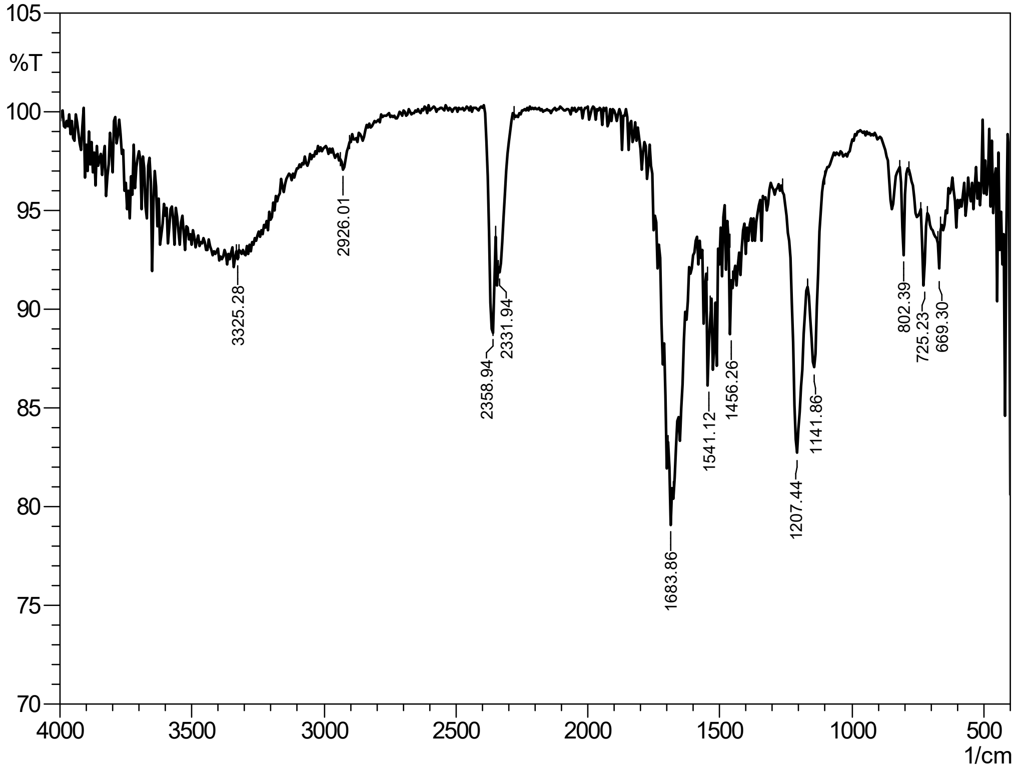

Figure S8. IR spectrum of **1**

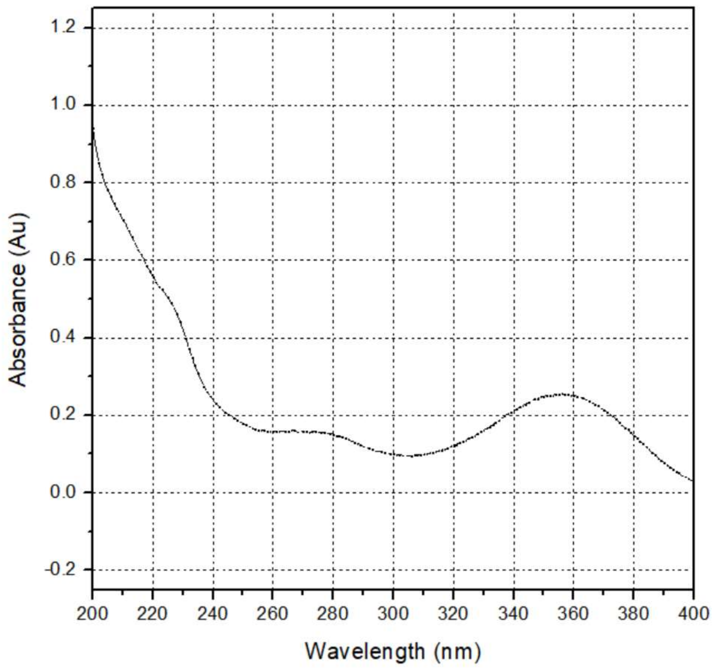

Figure S9. UV spectrum of **1**

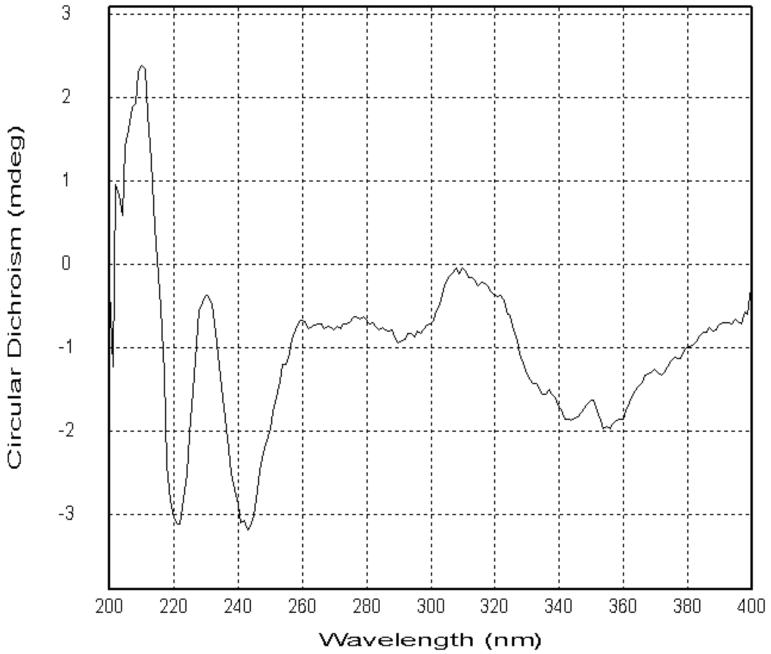

Figure S10. CD spectrum of **1**

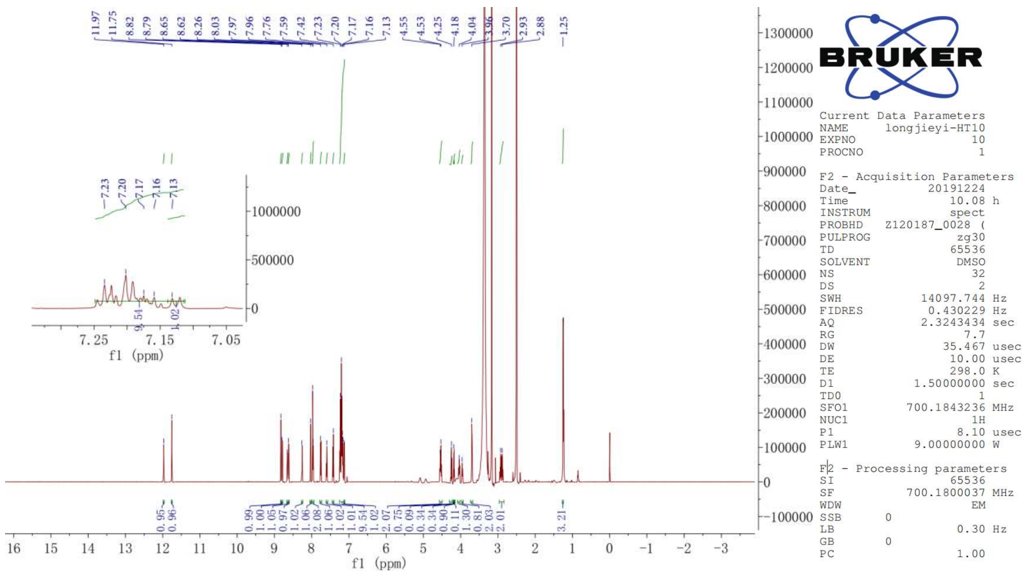

Figure S11. ^{1}H NMR spectrum of **2** (DMSO-d_6, 700 MHz)

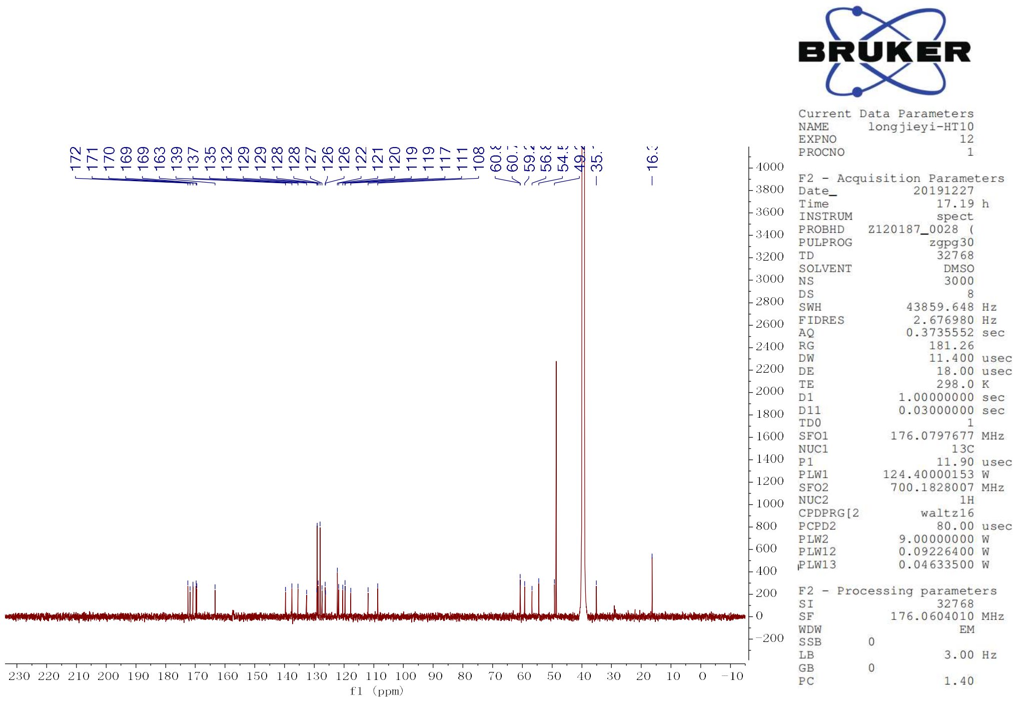

Figure S12. ^{13}C NMR spectrum of **2** (DMSO-d_6, 175 MHz)

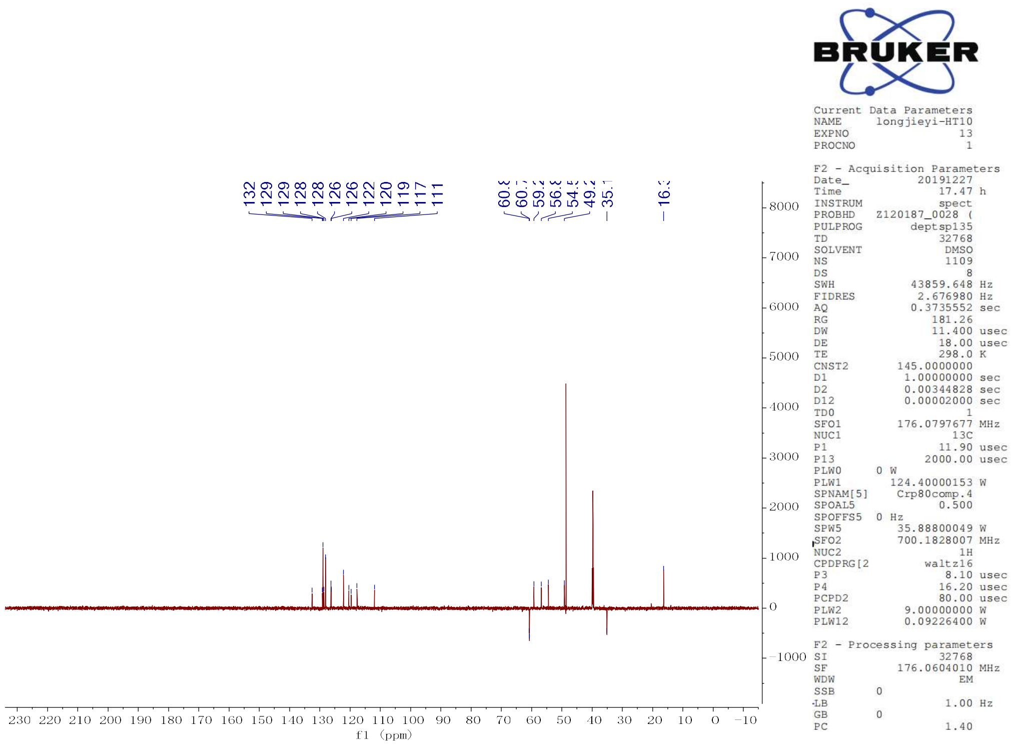

Figure S13. DEPT spectrum of **2** (DMSO-d_6)

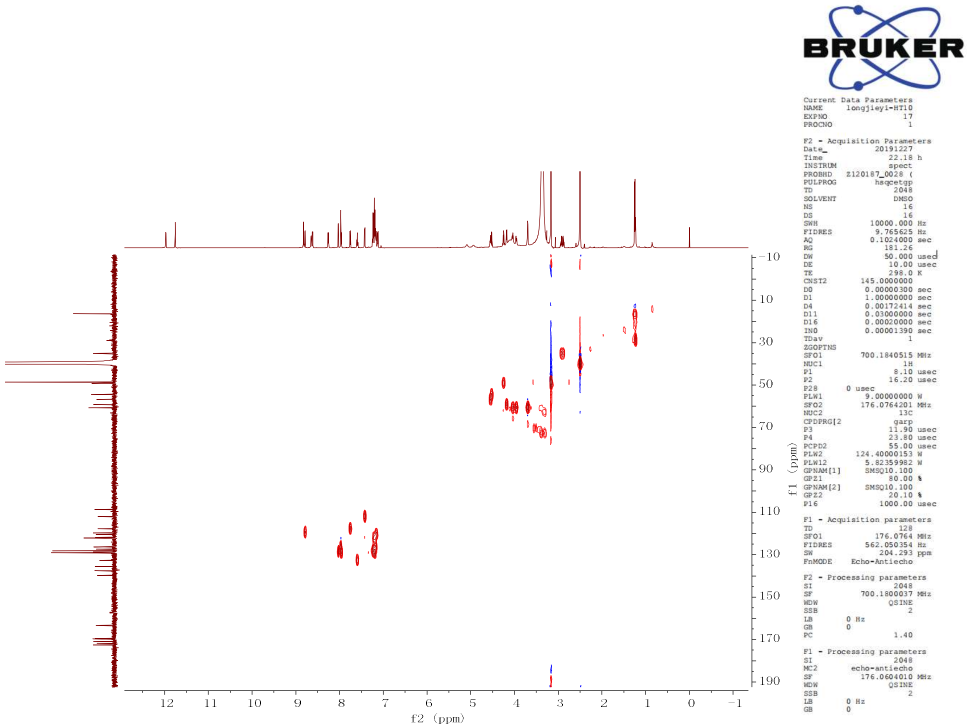

Figure S14. HSQC spectrum of **2** (DMSO-d_6)

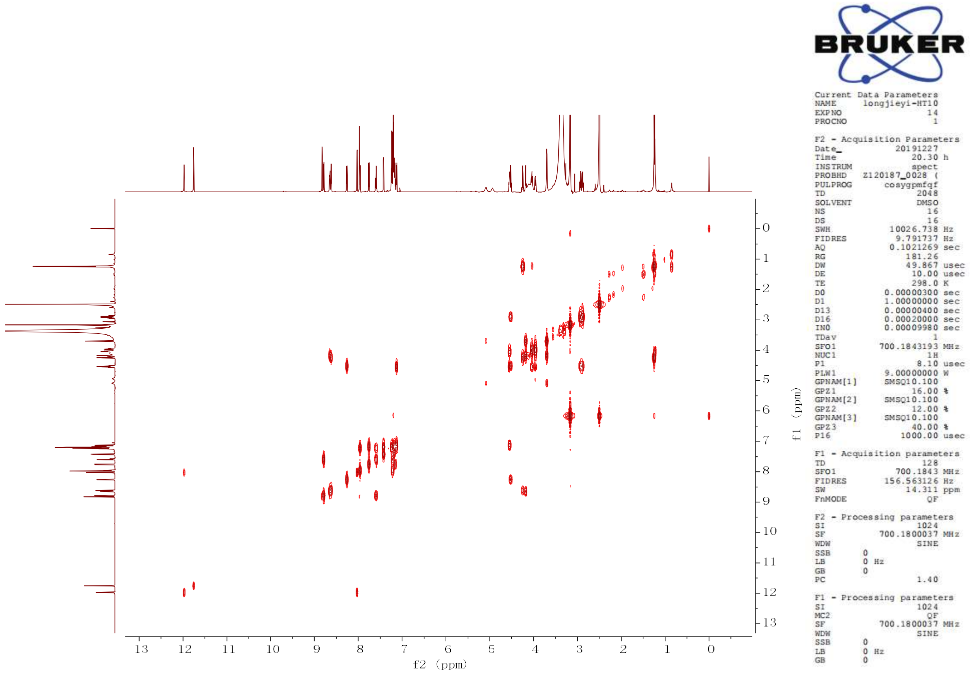

Figure S15. ^{1}H-^{1}H COSY spectrum of **2** (DMSO-d_6)

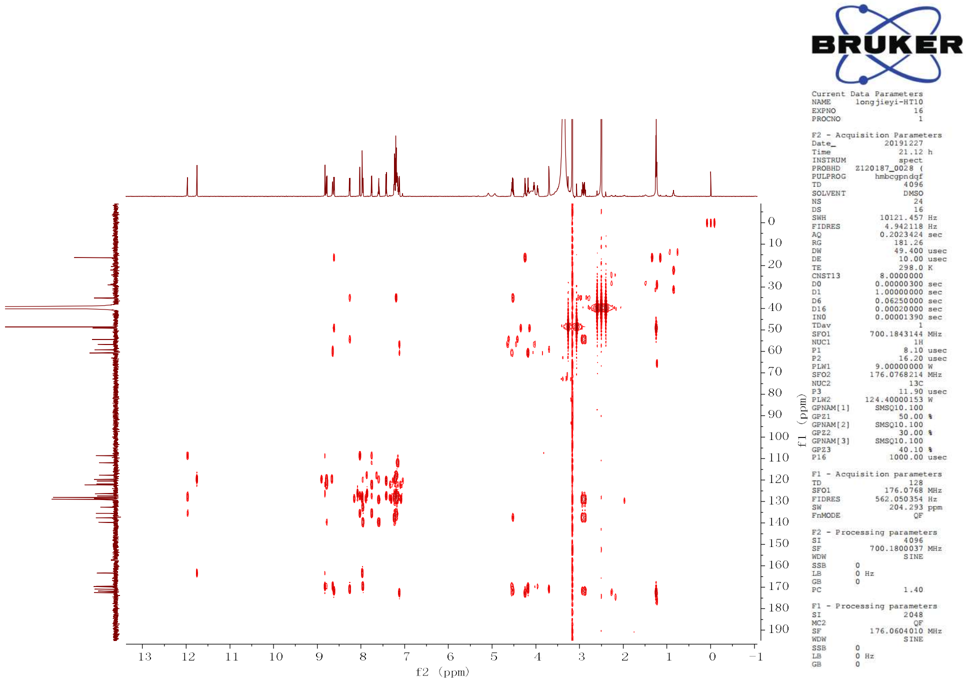

Figure S16. HMBC spectrum of **2** (DMSO-d_6)

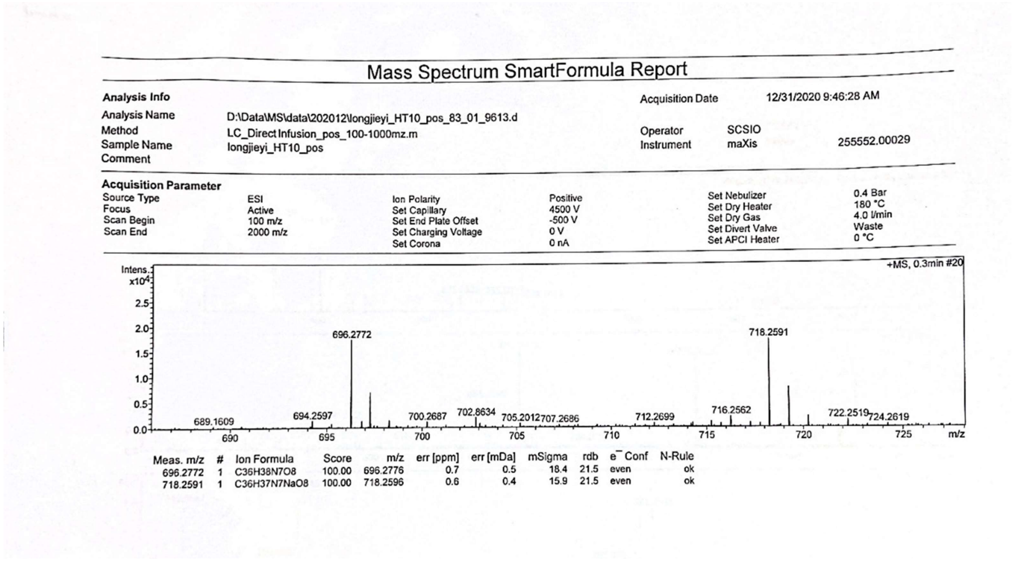

Figure S17. HRESIMS spectrum of **2**

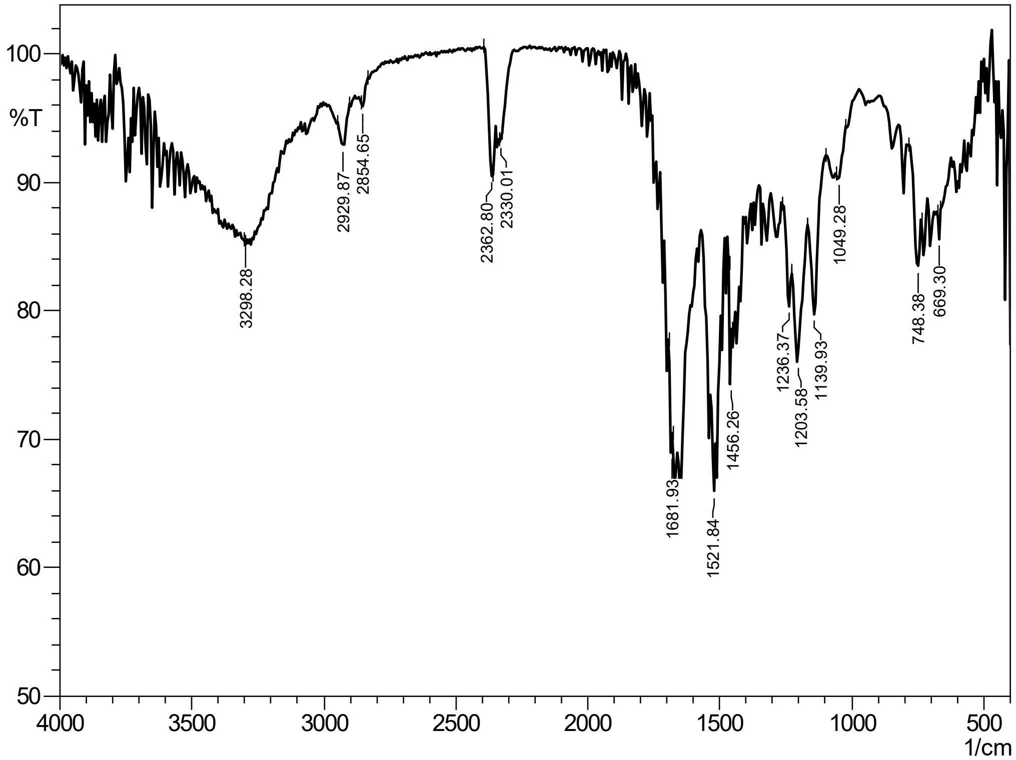

Figure S18. IR spectrum of **2**

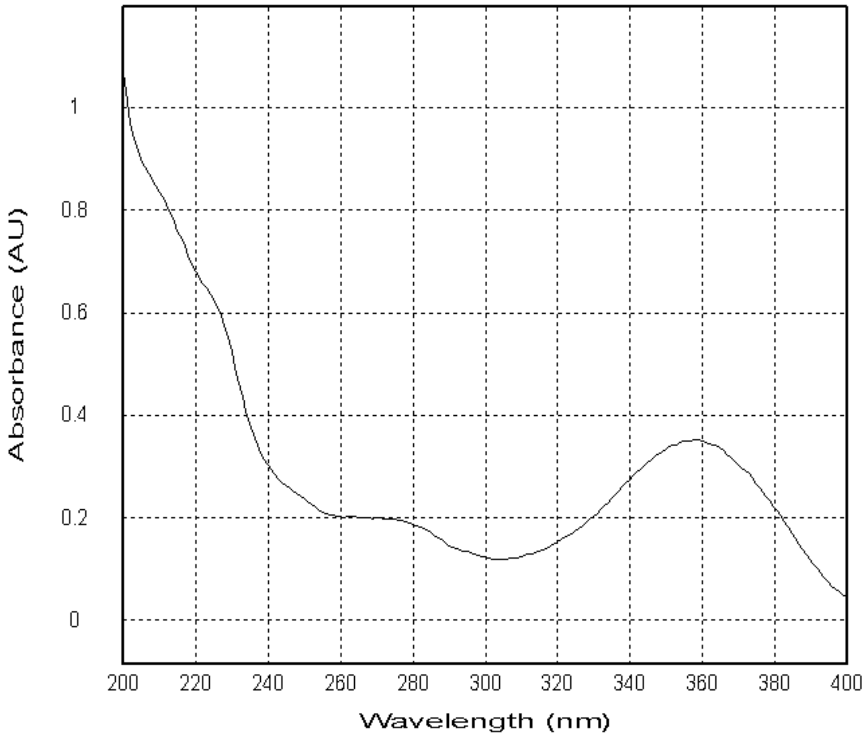

Figure S19. UV spectrum of **2**

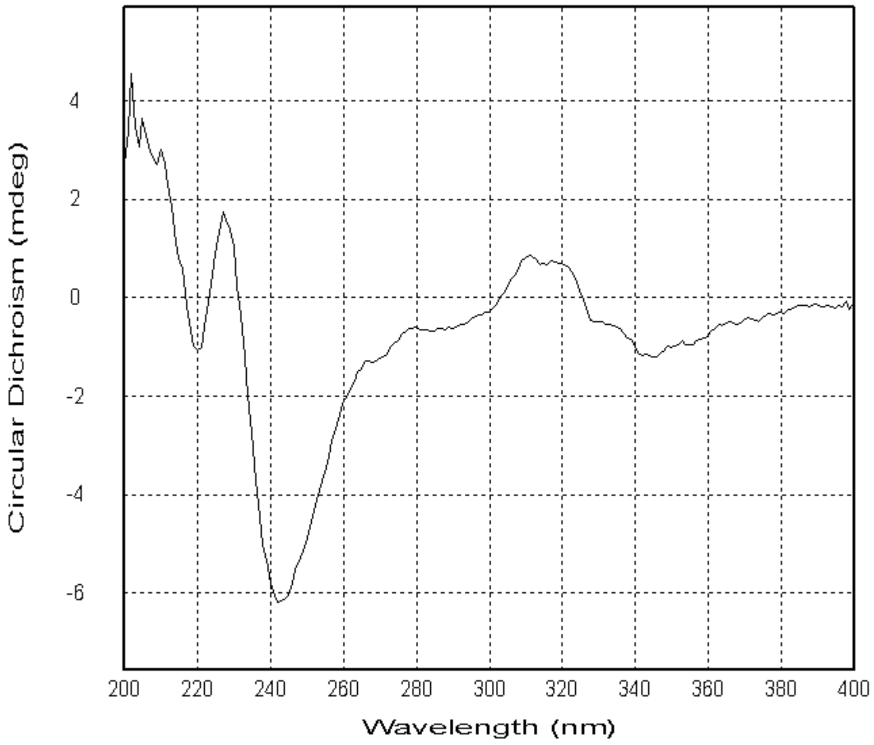

Figure S20. CD spectrum of **2**

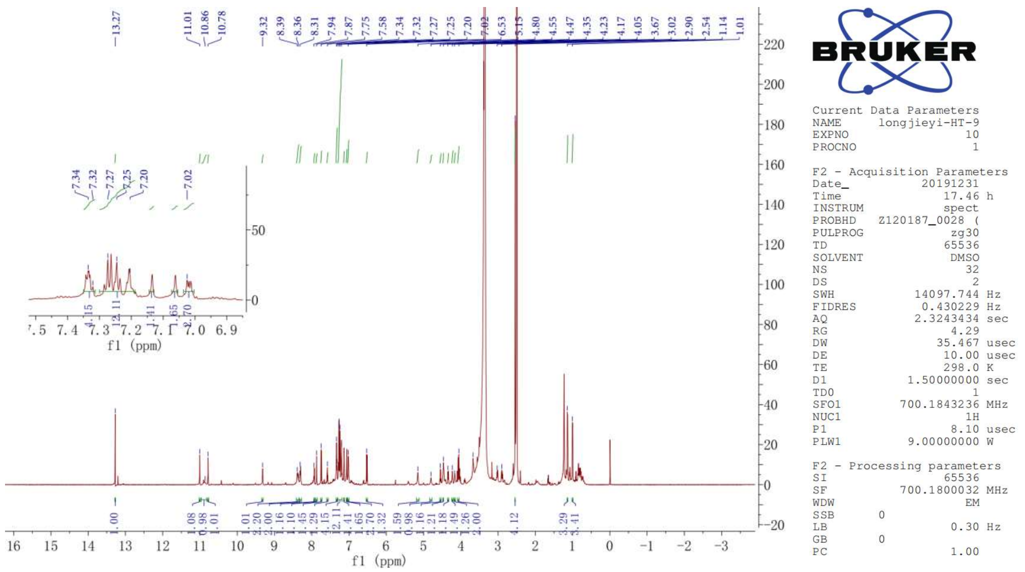

Figure S21. ^{1}H NMR spectrum of **3** (DMSO-d_6, 500 MHz)

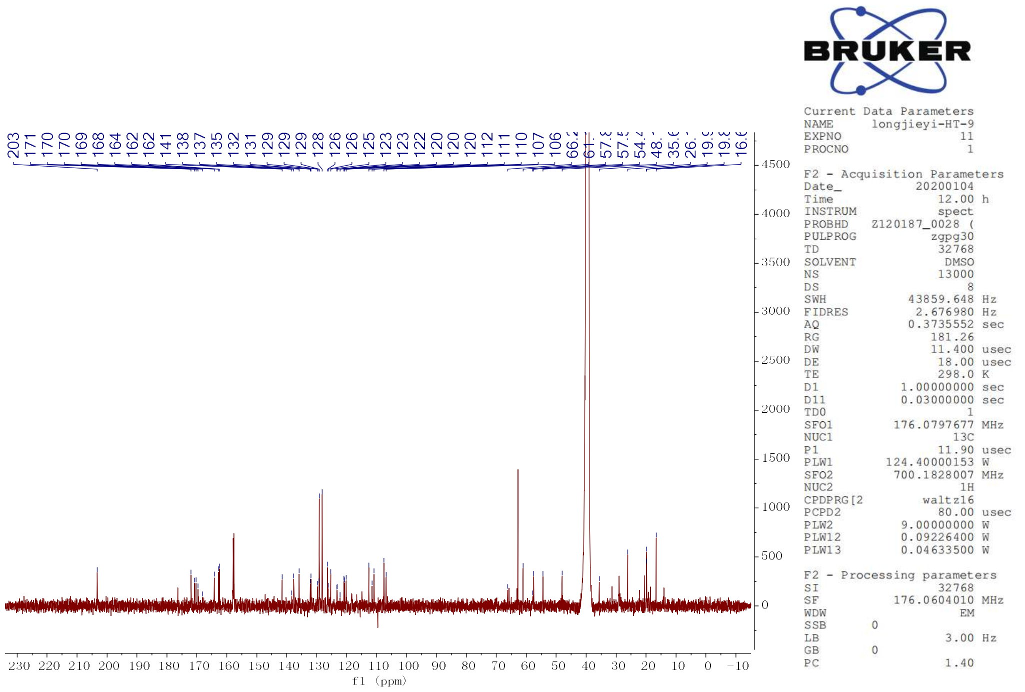

Figure S22. ^{13}C NMR spectrum of **3** (DMSO-d_6, 125 MHz)

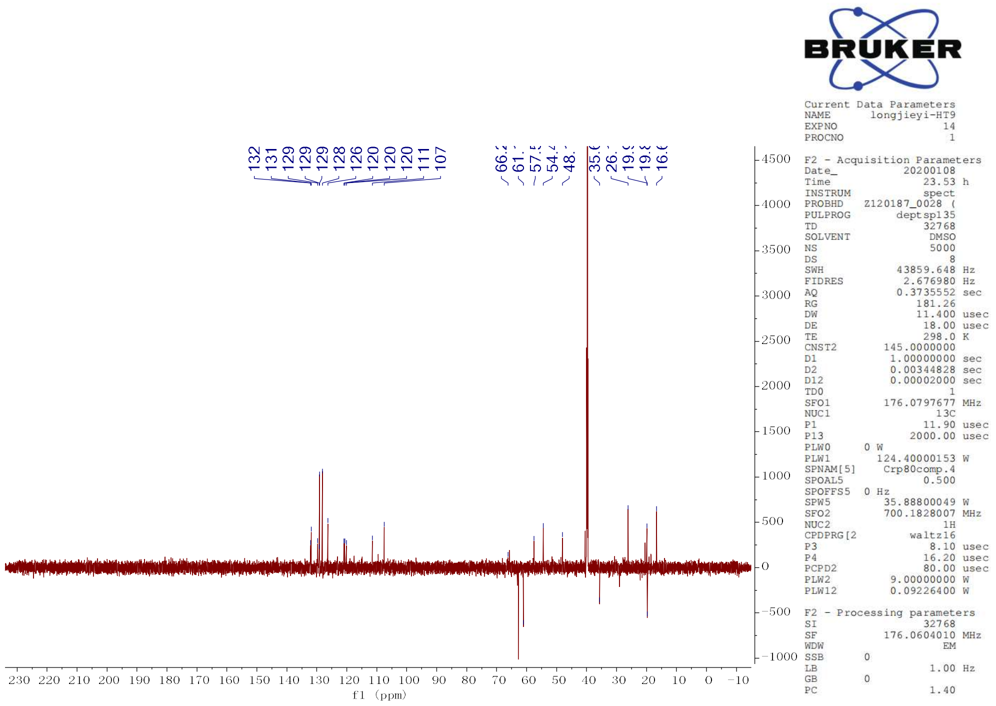

Figure S23. DEPT spectrum of **3** (DMSO-d_6)

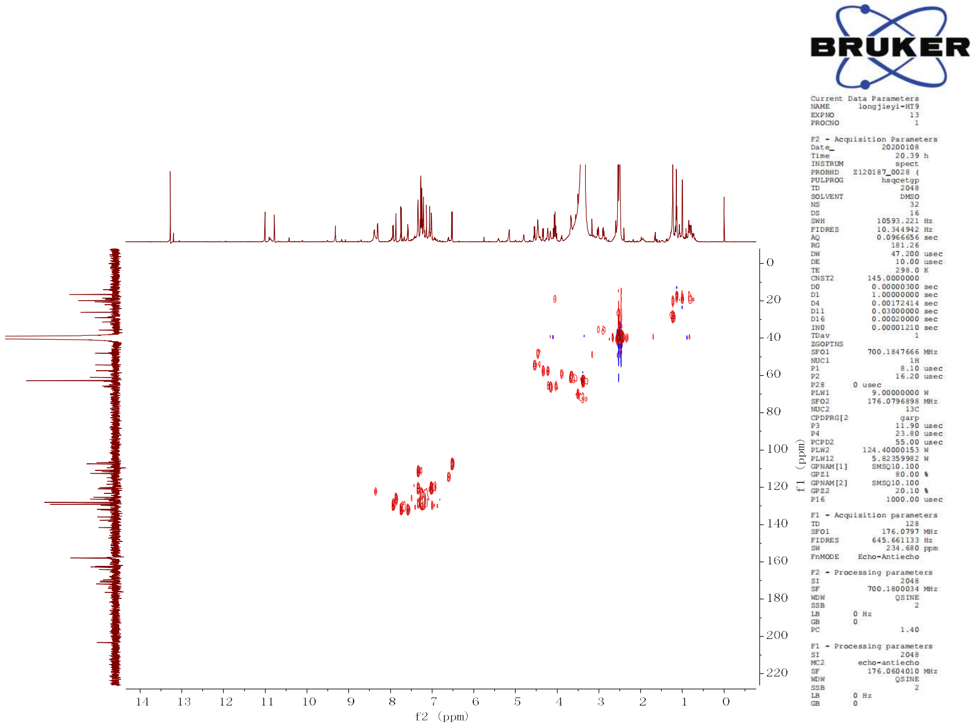

Figure S24. HSQC spectrum of **3** (DMSO-d_6)

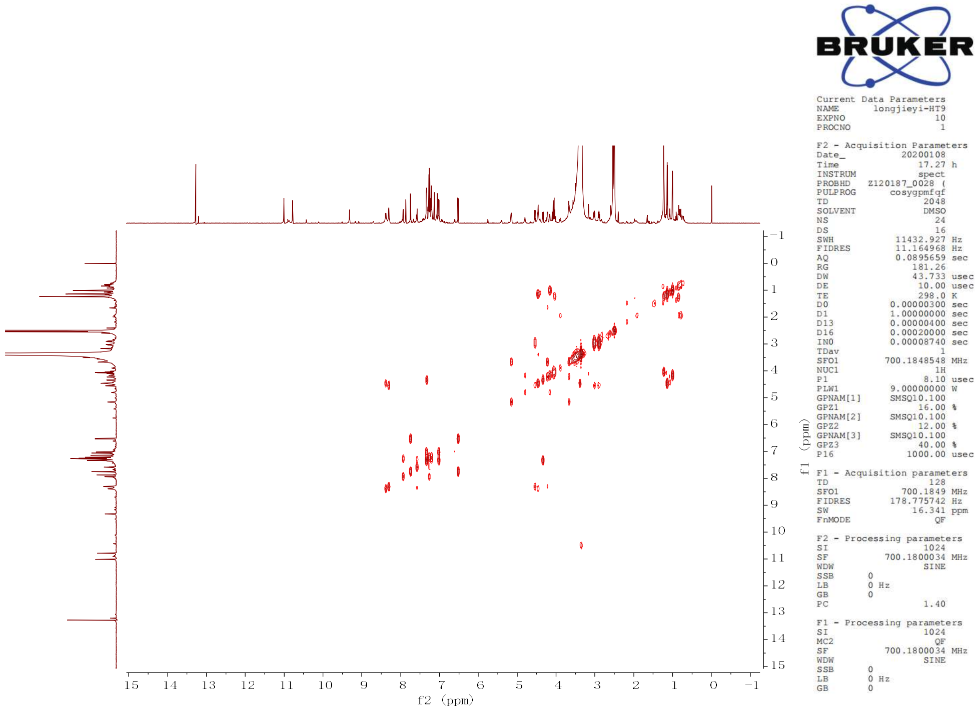

Figure S25. ^{1}H-^{1}H COSY spectrum of **3** (DMSO-d_6)

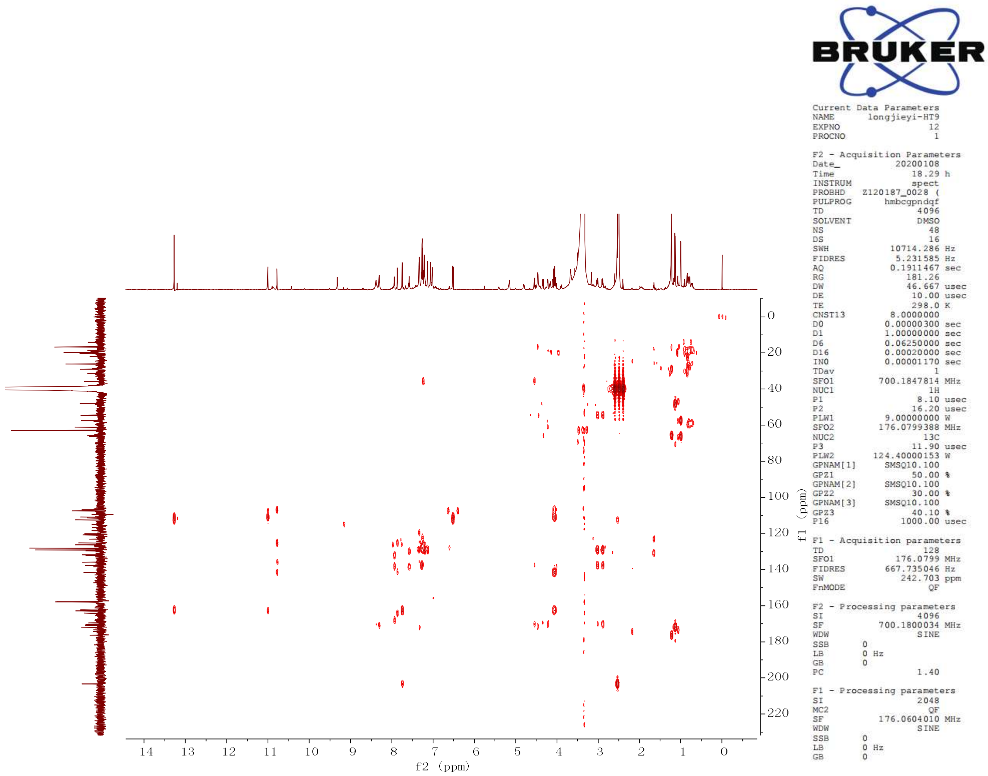

Figure S26. HMBC spectrum of **3** (DMSO-d_6)

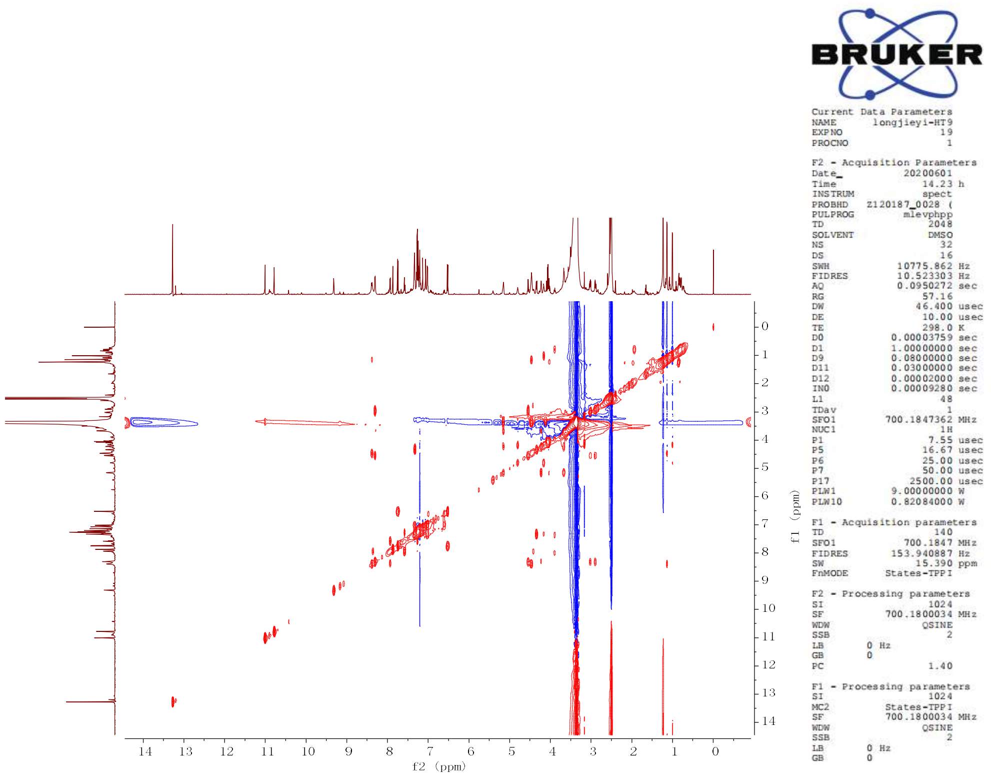

Figure S27. TOCSY spectrum of **3**

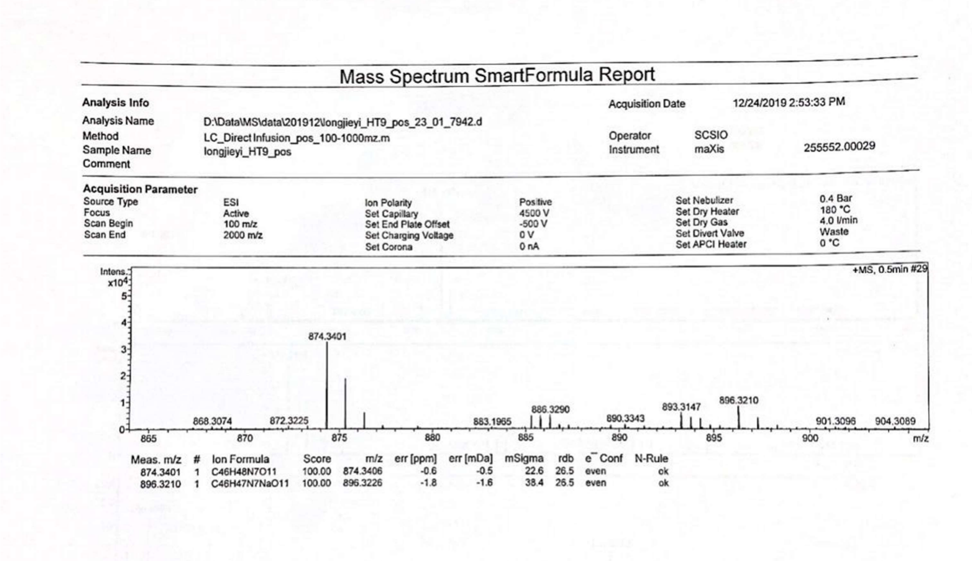

Meas. m/z	#	Ion Formula	Score	m/z	err [ppm]	err [mDa]	mSigma	rdb	e⁻ Conf	N-Rule
874.3401	1	C46H48N7O11	100.00	874.3406	-0.6	-0.5	22.6	26.5	even	ok
896.3210	1	C46H47N7NaO11	100.00	896.3226	-1.8	-1.6	38.4	26.5	even	ok

Figure S28. HRESIMS spectrum of **3**

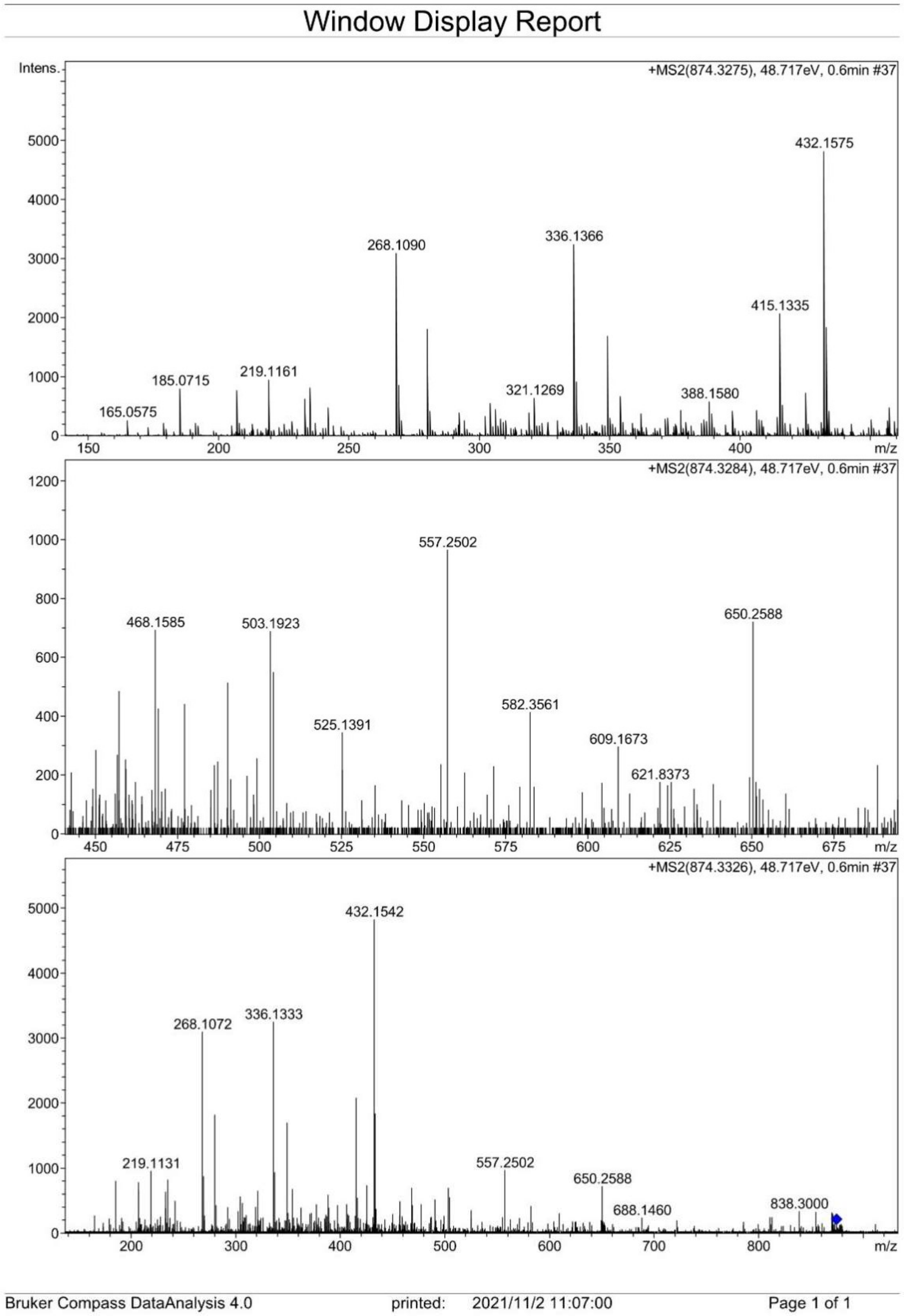

Figure S29. HRESIMS/MS fragmentation of **3**

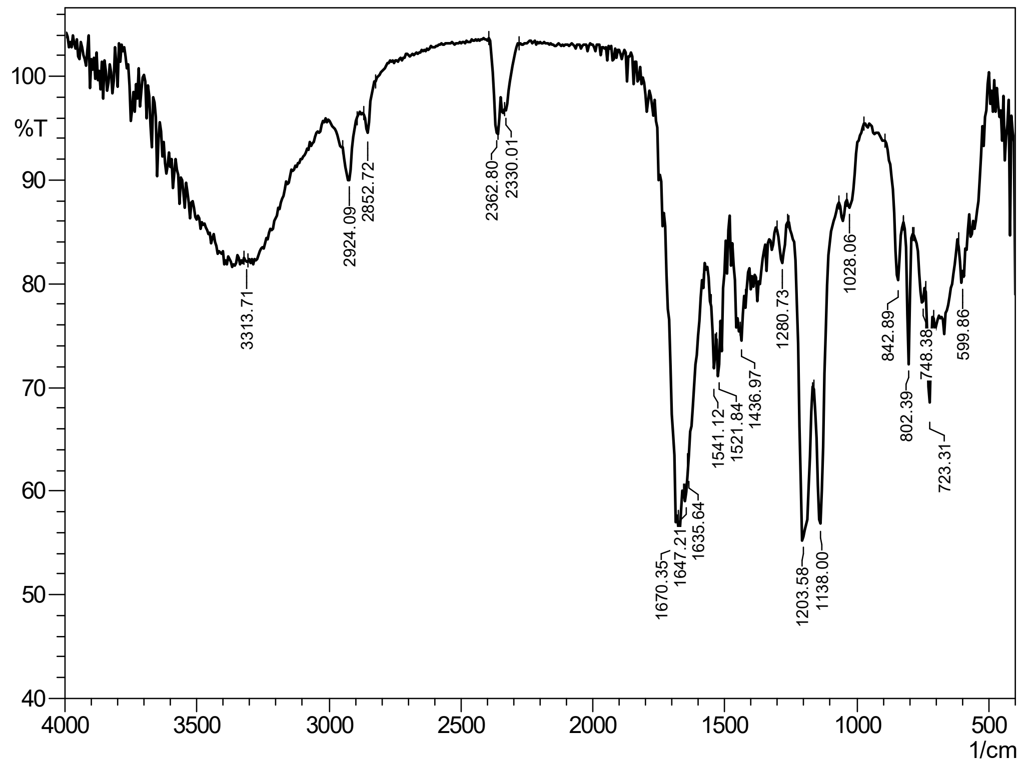

Figure S30. IR spectrum of **3**

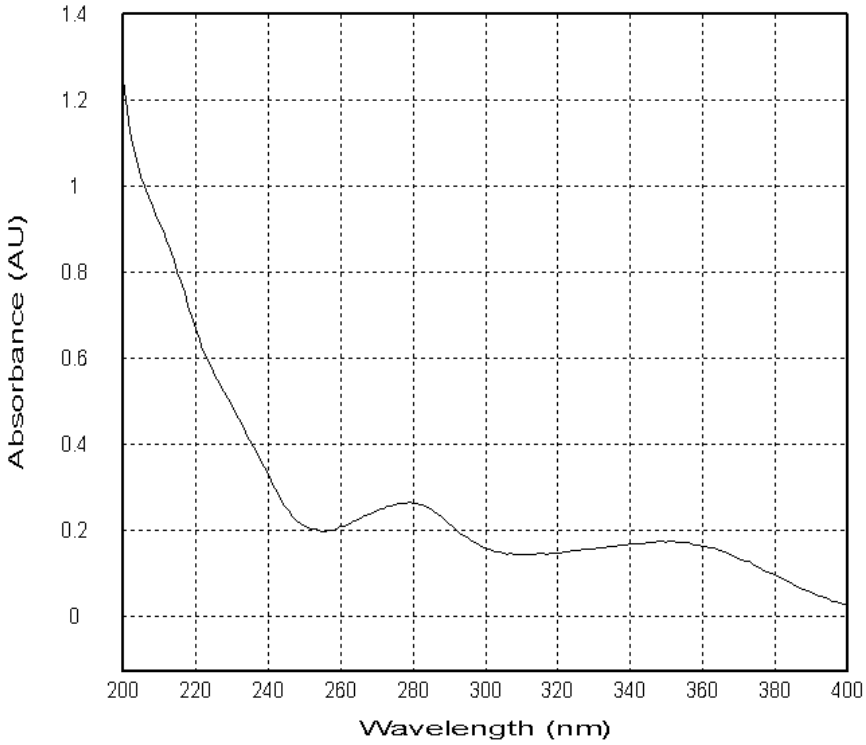

Figure S31. UV spectrum of **3**

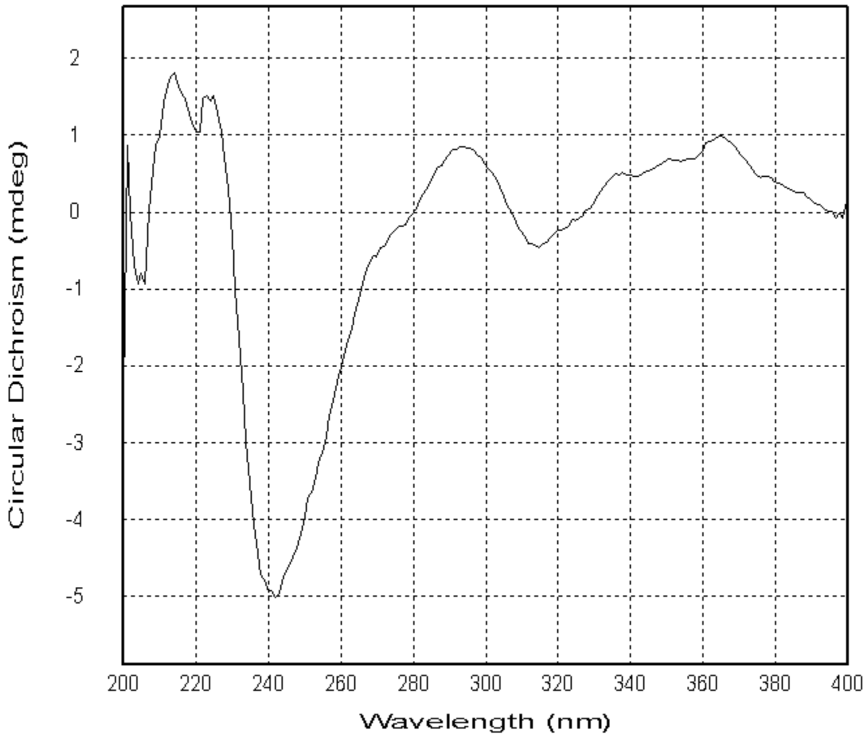

Figure S32. CD spectrum of **3**

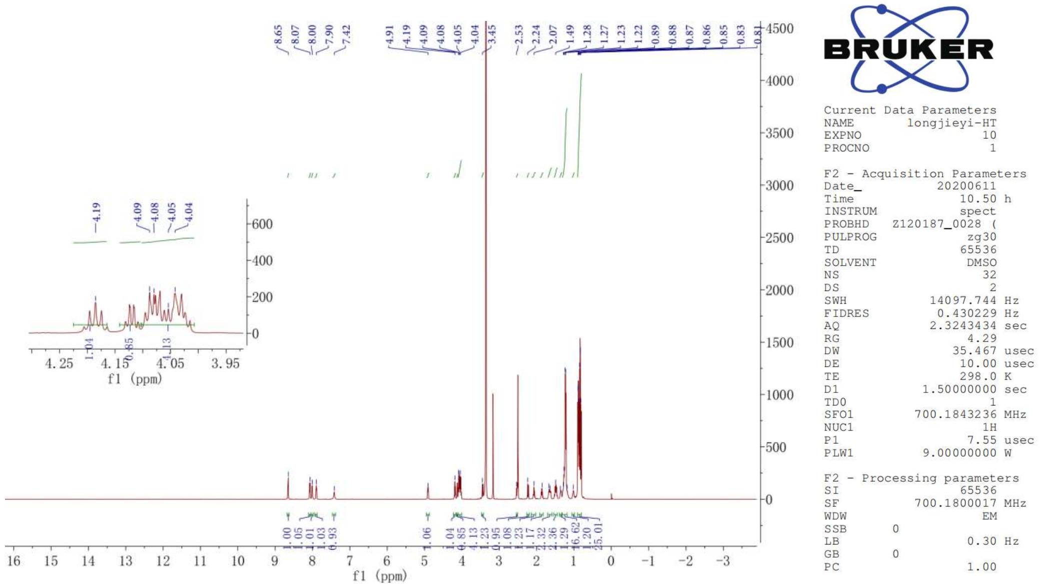

Figure S33. ^{1}H NMR spectrum of **4** (DMSO-d_6, 500 MHz)

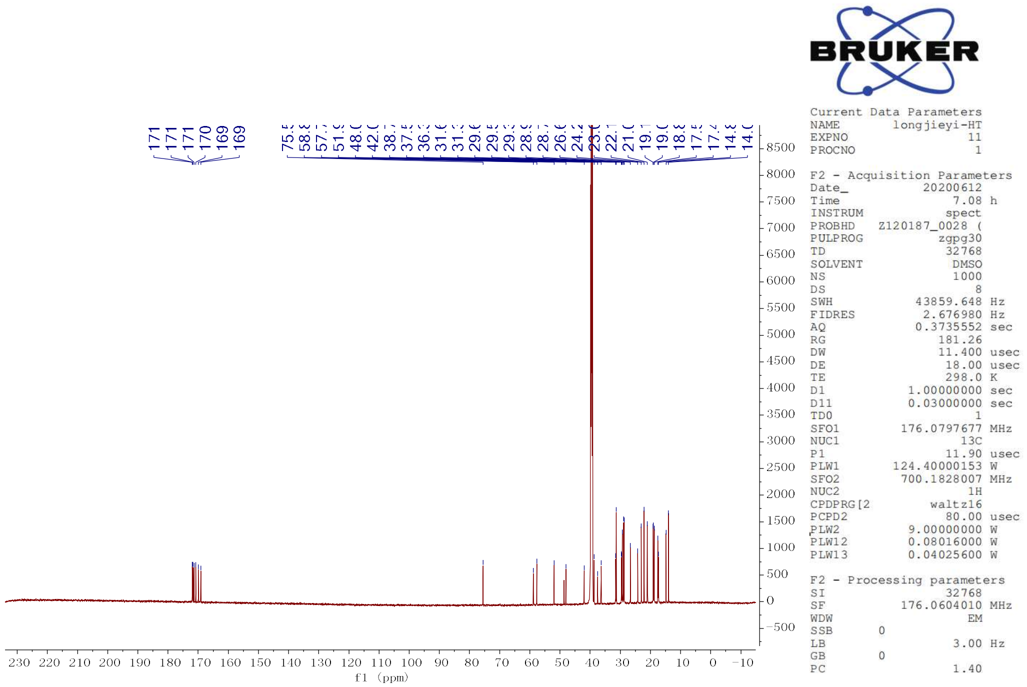

Figure S34. ^{13}C NMR spectrum of **4** (DMSO-d_6, 125 MHz)

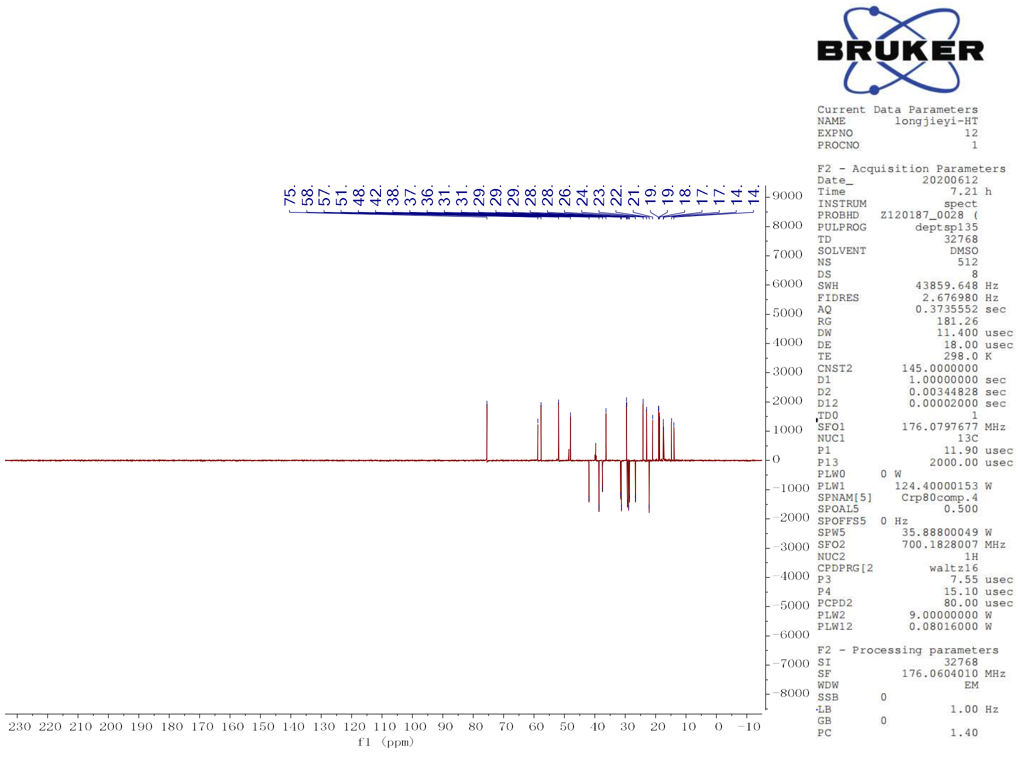

Figure S35. DEPT spectrum of **4** (DMSO-d_6)

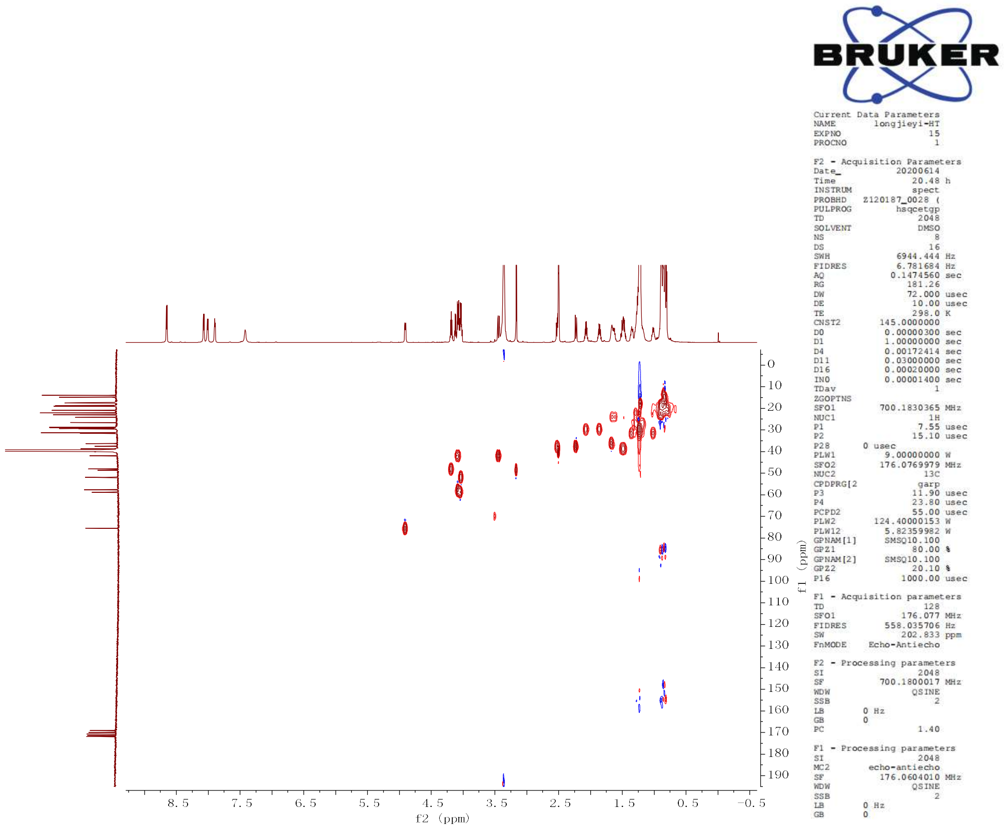

Figure S36. HSQC spectrum of **4** (DMSO-d_6)

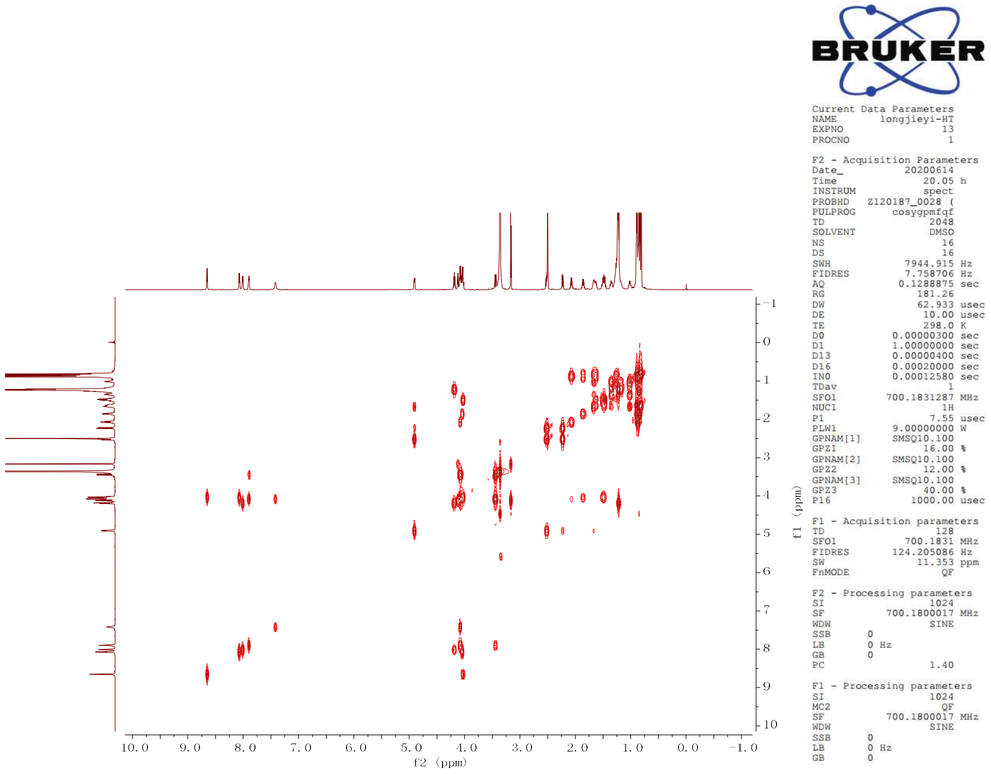

Figure S37. ^{1}H-^{1}H COSY spectrum of **4** (DMSO-d_6)

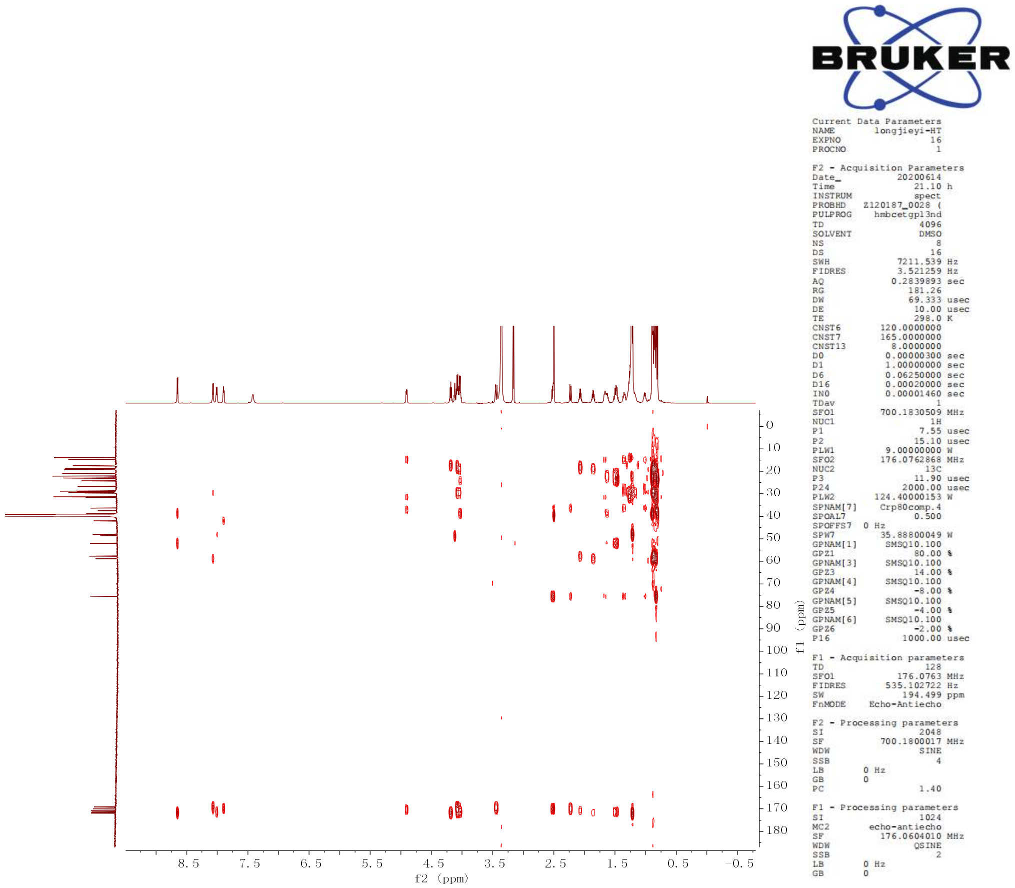

Figure S38. HMBC spectrum of **4** (DMSO-d_6)

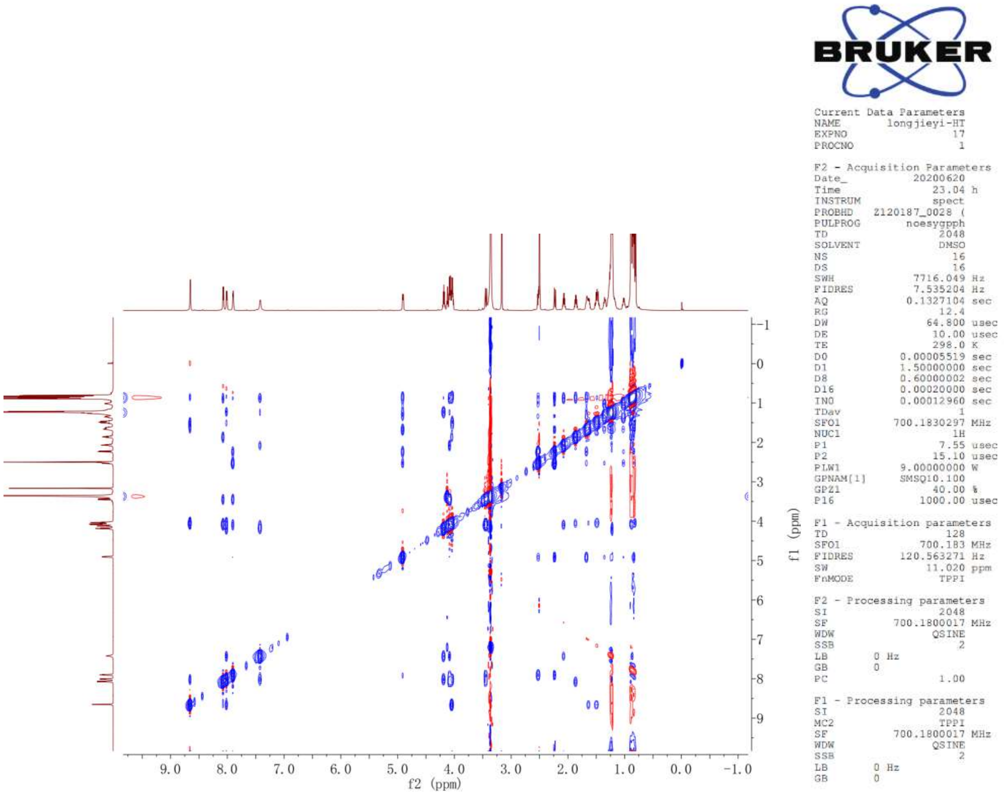

Figure S39. NOESY spectrum of **4** (DMSO-d_6)

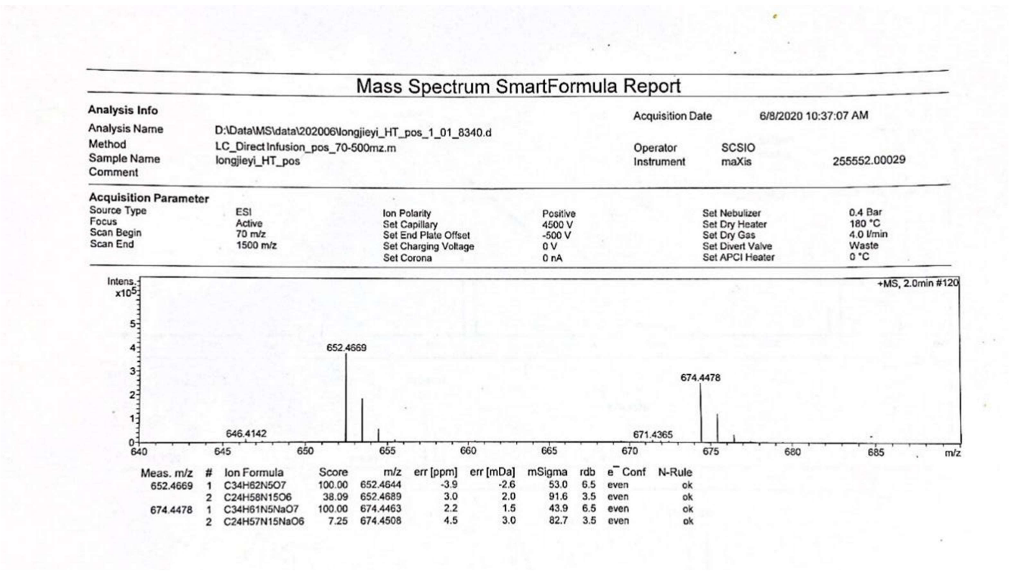

Figure S40. HRESIMS spectrum of **4**

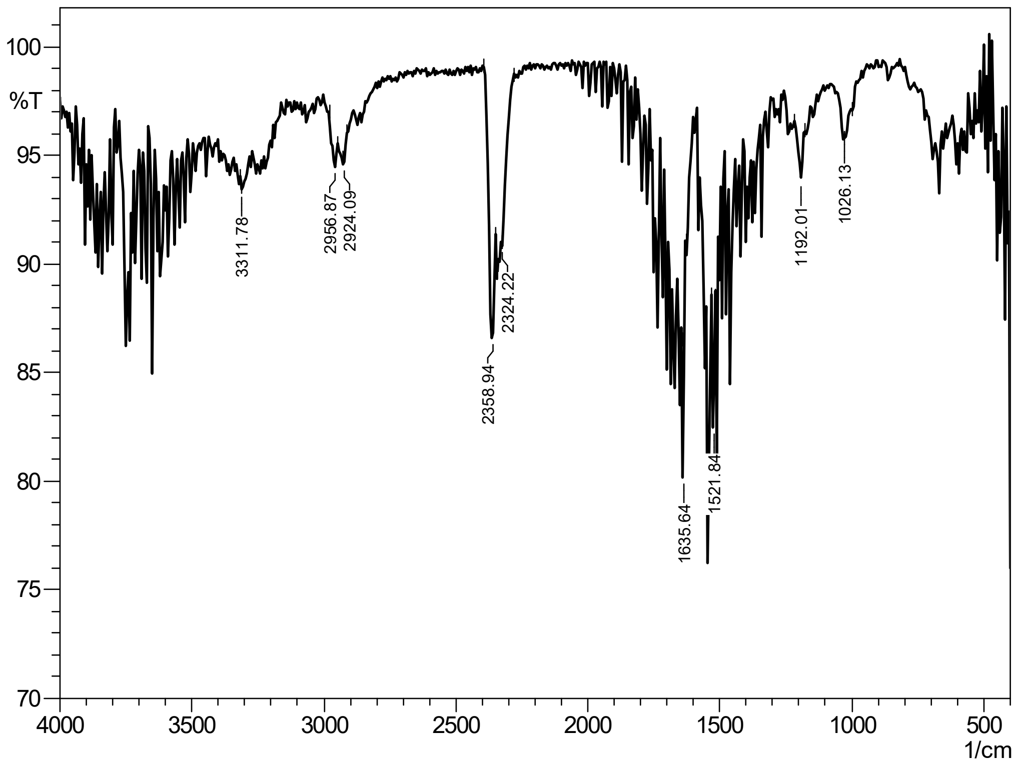

Figure S41. IR spectrum of **4**

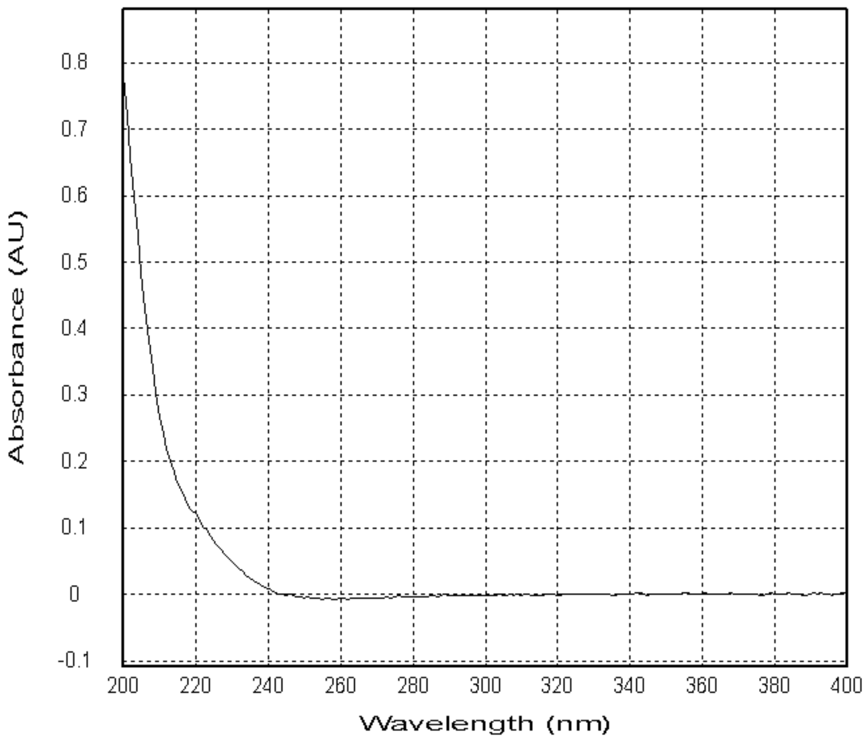

Figure S42. UV spectrum of **4**

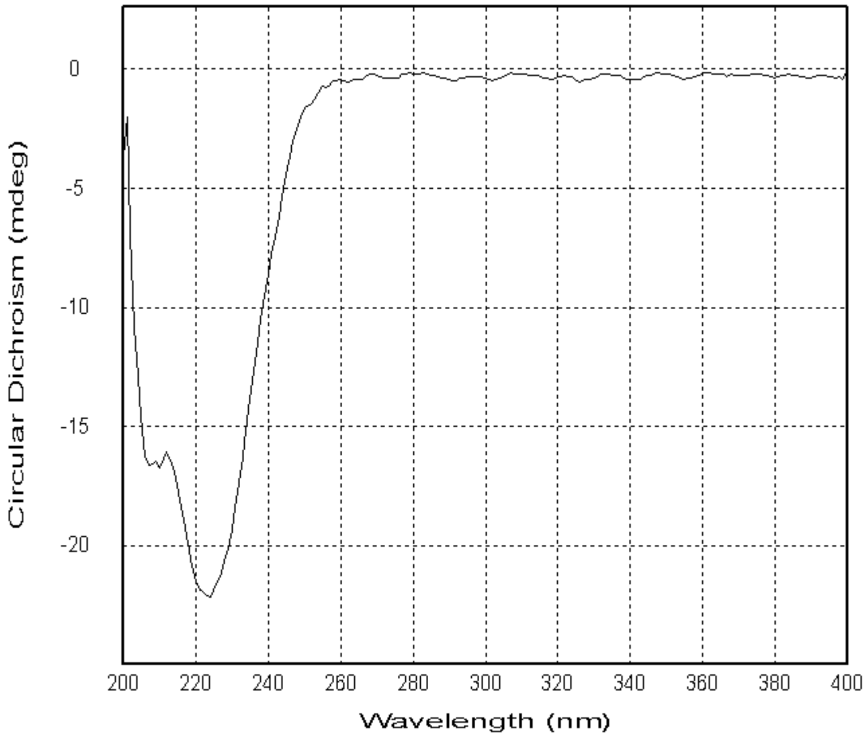

Figure S43. CD spectrum of **4**

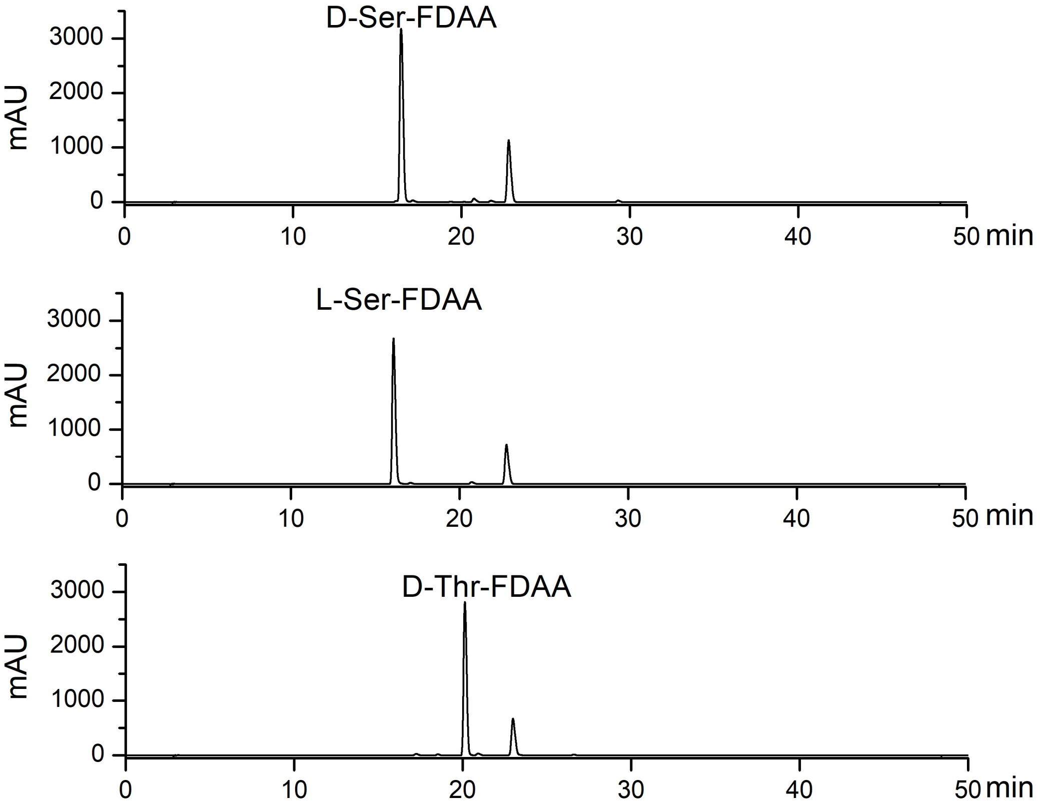

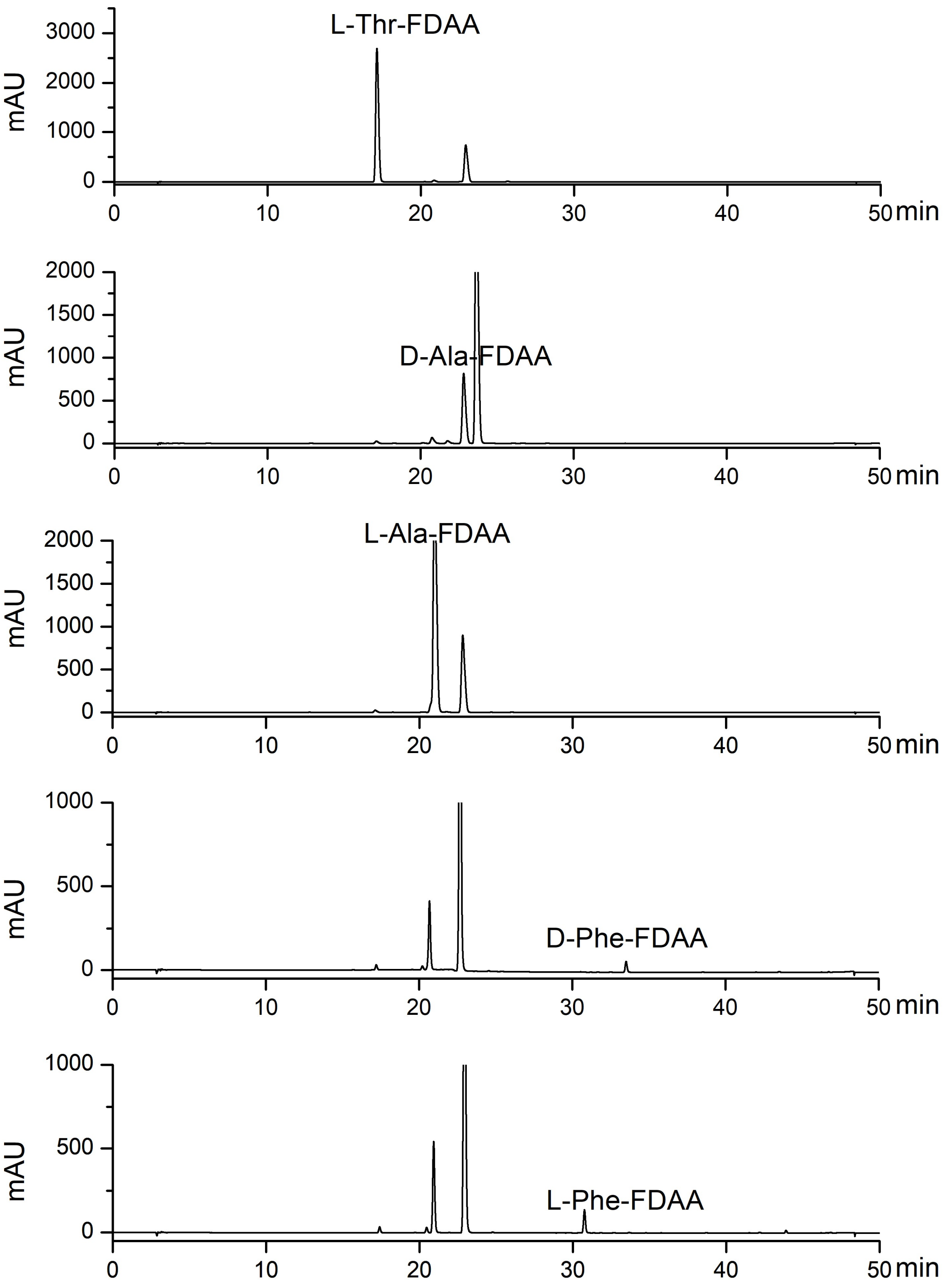

Figure S44. HPLC analysis of FDAA derivatives of standard amino acids (YMC-Pack ODS-A column, 250*4.6 mmI.D., S-5 μm, 12 nm).

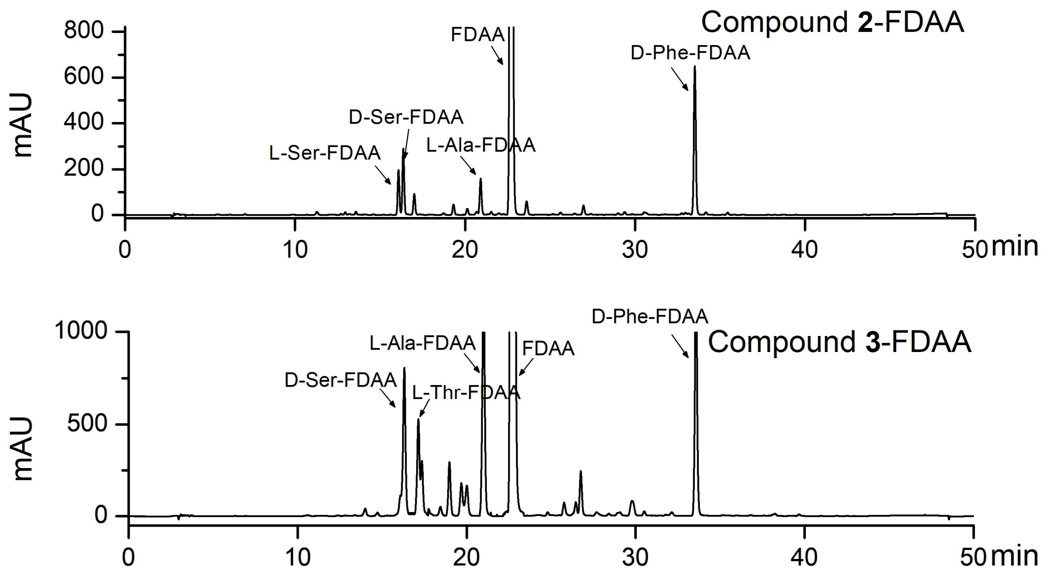

Figure S45. HPLC analysis of FDAA derivatives of compound **2** and **3** (YMC-Pack ODS-A column, 250*4.6 mml.D., S-5 μm, 12 nm)

Test consentration of 4 μg/mL	Average OD
100	0.54
75	0.56
50	0.61
25	0.71
13	1.62

Table S1.Test concentration and OD value of compound **4** against AChE.

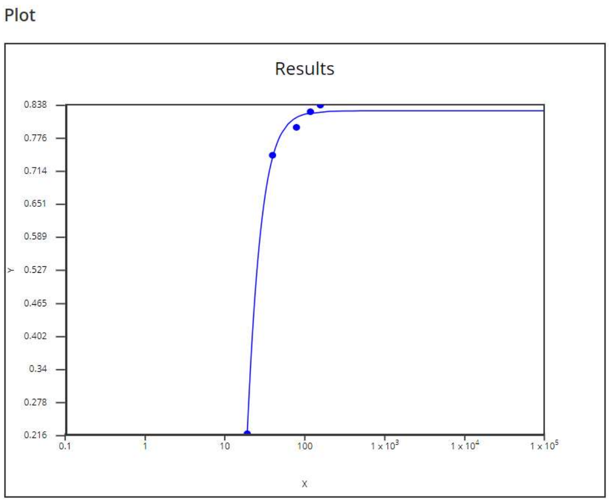

Figure S46. IC_{50} curve of compound **4** against AChE.

Test consentration of 4 μM	OD value of HONE1-EBV	Test consentration of 4 μM	OD value of HONE1
50.000	0.400	100.000	0.328
16.667	0.329	33.333	0.305
5.556	0.528	11.111	0.729
1.852	0.968	3.704	0.880
0.617	1.074	1.235	0.922
0.206	1.111	0.412	1.066
0.068	1.092	0.137	1.153
0.023	1.047	0.046	1.020
0.008	0.997	0.015	1.084
0.003	0.976	0.005	1.021

Table S2.Test concentration and OD value of compound **4** against HONE1-EBV and HONE1.

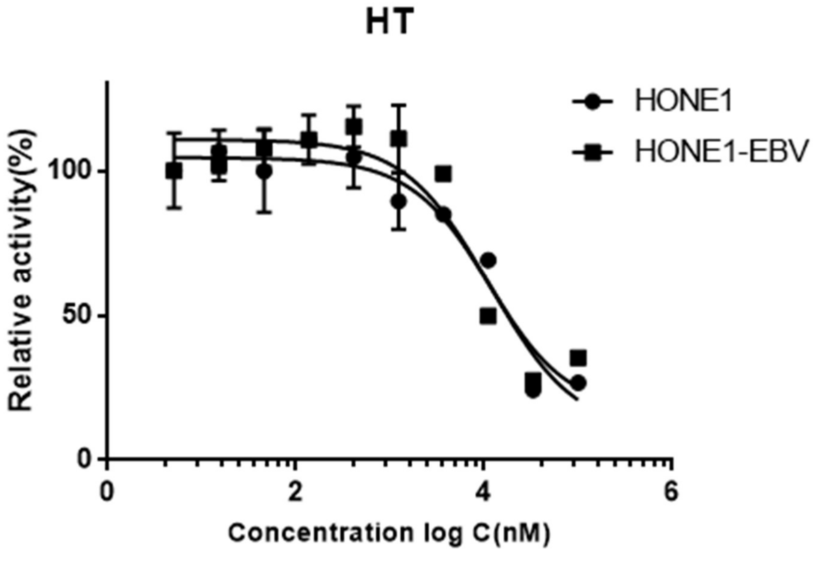

Figure S47. IC_{50} curve of compound **4** against HONE1-EBV and HONE1.
